# Combined analysis reveals a core set of cycling genes

**DOI:** 10.1186/gb-2007-8-7-r146

**Published:** 2007-07-24

**Authors:** Yong Lu, Shaun Mahony, Panayiotis V Benos, Roni Rosenfeld, Itamar Simon, Linda L Breeden, Ziv Bar-Joseph

**Affiliations:** 1 Department of Computer Science, Carnegie Mellon University, Forbes Avenue, Pittsburgh, Pennsylvania 15213, USA; 2 Department of Computational Biology, University of Pittsburgh Medical School, Lothrop Street, Pittsburgh, Pennsylvania 15213, USA; 3 Machine Learning Department, Carnegie Mellon University, Forbes Avenue, Pittsburgh, Pennsylvania 15213, USA; 4 Department of Molecular Biology, Hebrew University Medical School, Jerusalem, Israel 91120; 5 Basic Sciences Division, Fred Hutchinson Cancer Center, Fairview Avenue N, Seattle, Washington 98109, USA

## Abstract

**Background:**

Global transcript levels throughout the cell cycle have been characterized using microarrays in several species. Early analysis of these experiments focused on individual species. More recently, a number of studies have concluded that a surprisingly small number of genes conserved in two or more species are periodically transcribed in these species. Combining and comparing data from multiple species is challenging because of noise in expression data, the different synchronization and scoring methods used, and the need to determine an accurate set of homologs.

**Results:**

To solve these problems, we developed and applied a new algorithm to analyze expression data from multiple species simultaneously. Unlike previous studies, we find that more than 20% of cycling genes in budding yeast have cycling homologs in fission yeast and 5% to 7% of cycling genes in each of four species have cycling homologs in all other species. These conserved cycling genes display much stronger cell cycle characteristics in several complementary high throughput datasets.

Essentiality analysis for yeast and human genes confirms these findings. Motif analysis indicates conservation in the corresponding regulatory mechanisms. Gene Ontology analysis and analysis of the genes in the conserved sets sheds light on the evolution of specific subfunctions within the cell cycle.

**Conclusion:**

Our results indicate that the conservation in cyclic expression patterns is much greater than was previously thought. These genes are highly enriched for most cell cycle categories, and a large percentage of them are essential, supporting our claim that cross-species analysis can identify the core set of cycling genes.

## Abstract 2

The simultaneous analysis of expression data from multiple species reveals a core set of conserved cycling genes that is much larger than previously thought.

## Background

The cell cycle is a series of linked, fundamentally conserved processes that result in high-fidelity cell duplication. Global transcript levels throughout the cell cycle have been characterized using microarray expression data in several species. These include humans [1], budding and fission yeast [2-6], plants [7], and bacteria [8]. Early analysis of these experiments focused on individual species. Hundreds of genes have been identified whose transcripts oscillate during the cell cycle, and in budding yeast it is estimated that 15% of all genes are subject to this type of control. Despite this large cross-species effort, a number of studies have concluded that a surprisingly small number of genes conserved in two or more species are periodically transcribed in these species. Rustici and coworkers [4] compared fission and budding yeast expression data. Dyczkowski and Vingron [9] compared three lists of cycling genes (budding and fission yeast and human), and Jensen and colleagues [10] added a fourth species (*Arabidopsis*). All three studies concluded that periodicity at the transcript level was conserved across species in only a small number of cases.

When comparing cyclic expression patterns across species, researchers face several challenges. In some cases the lists derived for each species were generated using different expression analysis methods. For example, the scoring methods used by Spellman [2] and Rustici [4] and their colleagues are different, which makes direct comparison problematic. Another challenge arises when determining the set of homologs between the species being analyzed. Although using curated databases results in a more accurate set of conserved pairs, this analysis is limited to a small (and sometimes biased) set of genes. In addition, the binary assignment (ortholog or not) in databases cannot account for more complex similarity measures, which are often represented using a more continuous value (for example, BLAST e-value). Relying on the actual strength of homology may help when looking for conserved sets. Finally, expression data are noisy. Repeated experiments, even within the same species, often result in relatively low agreement [5], and differences between species may be even more problematic because radically different synchronization procedures must be used [11]. Any combination of the above may bias the analysis and prevent the identification of an accurate set of conserved cycling genes.

Here we use an algorithm that analyzes data from all species concurrently. This differs from previous methods that performed this analysis separately for each species and then looked at the overlap. Our method overcomes many of the obstacles discussed above. We use the same scoring method for all species, and include parameters that allow a gene in one species to influence the score of a homologous gene (in either the same or in another species). These parameters are continuous and depend on the similarity between the genes. They allow for one to many and for many to many mappings between genes; they also allow higher quality expression data in one species to improve the quality of the data for other species.

We analyze expression data from four species: budding [2] and fission yeast [4-6], human [1], and plants [7]. Our primary goal is to determine sets of genes that are conserved in sequence and at the transcript level between all and subsets of these species. Our findings indicate that the set of conserved cycling genes is much larger than was previously thought. These findings are validated and explained using a large number of complementary high throughput datasets.

## Results and discussion

### Combined analysis of cell cycle expression data

**Figure 1:**
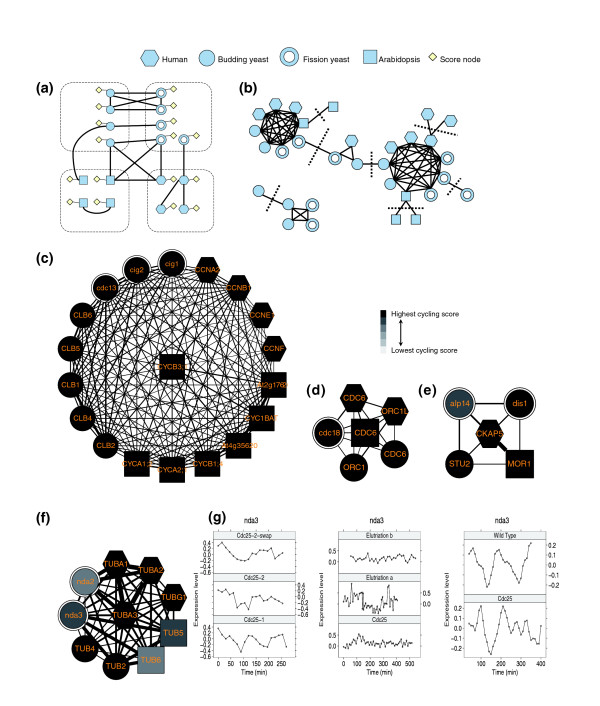
Method overview. **(a) **Genes (nodes in the graph) are connected to other genes based on sequence similarity. Species identity is indicated by shape of nodes. Genes are also connected to a 'score node', which represents cycling expression score. Information is propagated along the edges until convergence. Genes are assigned a posterior score and a cut-off is applied to select the top genes for each species. **(b) **The subgraph containing the selected genes is further analyzed by identifying multidomain homology cliques. Examples of identified cliques of conserved genes are presented in panels c to f. **(c) **Cyclins. Fission yeast Cig2 promotes the onset of S phase [45]. Human Ccna2 is part of the G2 checkpoint [46]. **(d) **Cdc6/Cdc18 is a conserved and essential component of pre-replication complexes (pre-RCs). Orc1 is the largest subunit of the origin recognition complex (ORC), which binds specifically to replication origins and triggers the assembly of pre-RCs [47]. **(e) **TOG related proteins, a family of microtubule-associated proteins (MAPs). Proteins in this group localize to the plus-end tips of microtubules and are essential for spindle pole organization. Alp14 is a component of the Mad2-dependent spindle checkpoint cascade sharing redundant functions with Dis1. Mutants with both genes knocked out are nonviable [48]. **(f,g) **Microtubule component clique and expression profiles for fission yeast Nda3 in eight experiments [4-6]. Nda3, a known cell division gene [49], obtains a high cycling score but is not one of the 600 top cycling fission genes based on expression analysis. Using our method, its score is correctly elevated because its sequence similarity to high scoring genes.

We developed an algorithm for combining sequence and expression data in order to identify cycling genes [12]. The algorithm uses probabilistic graphical models, and in particular Markov random fields, to combine these data sources. Genes are represented as nodes in the graph and are connected by edges to other genes (in the same species and all other species), based on their sequence similarity as determined by a BLAST score (Figure 1a). Each node (gene) is assigned an initial cycling score that is determined from expression data using a method from de Lichtenberg and coworkers [13]. Starting with this score, we propagate information along the edges of the graph until convergence. Thus, if a node with a medium to high score is connected to a set of nodes with high scores, then the information from the neighboring nodes can be used to elevate our belief in the assignment of this node, and *vice versa*. This method allows us to identify several cycling genes that can be missed in an analysis focused on a single species as a result of expression noise (Figure 1 and Additional data file 1 [Supporting Figures 1 to 3]). Similarly, genes with marginal scores that are only connected to low scoring genes can be filtered out of the cycling gene lists. Once the algorithm converges each gene is assigned a posterior cycling score between 0 and 1. For comparison reasons, we select for each species a set of genes with roughly equal size to those used in the original reports (although the identity of these genes is different), remove all other genes from the graph, and consider only the subgraph induced by the selected genes. This graph is analyzed to identify multidomain homology cliques [14] (Figure 1b-e). Each of these cliques is then analyzed to determine the set of species included. These findings are reported as three cell cycle conservation (CCC) sets with conservation across two (budding and fission yeast), three (yeasts and human cells), or all four species.

See Materials and methods and Additional data file 3 for further details on our graph-based algorithm and on clique analysis. Also see our supporting website [15] for a complete list of genes identified using our algorithm.

### Analysis of identified cycling genes

Our method combines expression and sequence data. This raises an obvious question; is the quality of our lists comparable to the quality of previous lists that relied on expression data alone? In other words, does our method sacrifice the accuracy with respect to the set of cycling genes in each species in order to obtain a larger set of conserved genes?

**Figure 2:**
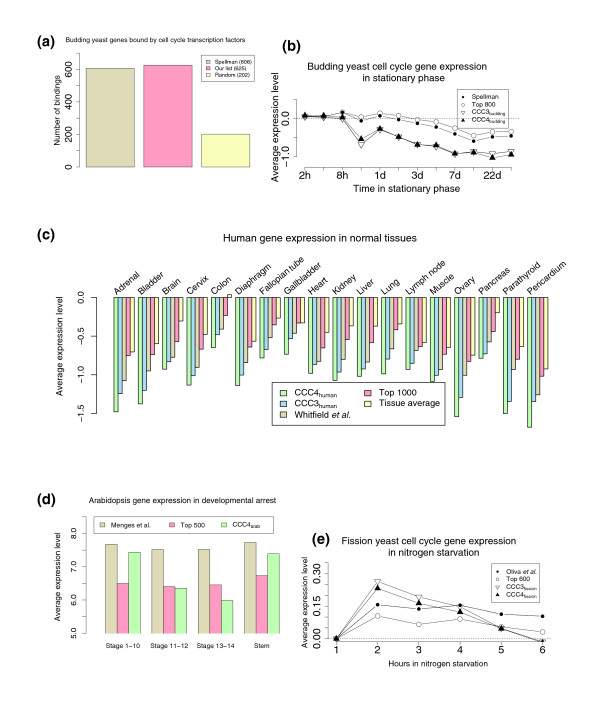
Analysis of cycling genes using complementary high throughput datasets. **(a) **Number of interactions between cycling genes and nine cell cycle transcription factors. **(b) **Average expression level of sets of budding yeast genes in stationary phase (data from Gasch and coworkers [50]). **(c) **Expression levels of human genes in normal tissues, using data presented by Shyamsundar and colleagues [51] (also see Additional data file 1 [Supporting Figure 4]). Genes in the conserved set have lower expression levels for most nonproliferating normal tissues when compared with the full list and the list presented by Whitfield and coworkers [1]. For 26 out of 36 normal tissues this difference is significant with a *P *value < 0.05. **(d) ***Arabidopsis *cells in developmental arrest experiments [52]. Flower cells in the mutants stop growing after stage 11, whereas cells in the stem grow normally. Again, the conserved set is expressed at lower levels in developmental arrest (*P *= 0.027 at stages 11 and 12; *P *= 0.003 at stages 13 and 14). **(e) **Expression data from studying sexual differentiation and mating in fission yeast [53].

A possible way to assess the quality of such lists is by comparing them with other high-throughput data sources [13]. For example, protein-DNA binding data are available for nine budding yeast transcription factors that are known to be involved in cell cycle specific transcription [16]. It is expected that many cycling genes would be bound by these factors. When comparing the genes in our list with the original list [2], we find that both exhibit a threefold enrichment for these interactions compared with a random gene list (Figure 2a). Stationary phase expression experiments yield similar results (Figure 2b). Similarly, both our list and the original list [1] of cycling human genes are enriched for binding of known cell cycle factors (Nrf1 and E2f2; Additional data file 1 [Supporting Figure 4d]). Genes on both lists exhibit lower expression levels in nonproliferating tissues (Figure 2c) and higher expression levels in cancer cells (Additional data file 1 [Supporting Figure 4c]). Expression data for fission yeast and *Arabidopsis *support our list for these species as well (Figure 2d-e).

Combined, these results indicate that the species-specific lists derived using our method are comparable in quality to those of previously reported cell cycle gene lists. Additional data file 2 (Supporting Tables 1 to 3) presents the percentage overlap between the lists of cycling genes identified using our method and previously reported cycling gene lists for the four species.

### Conserved cycling genes

**Figure 3:**
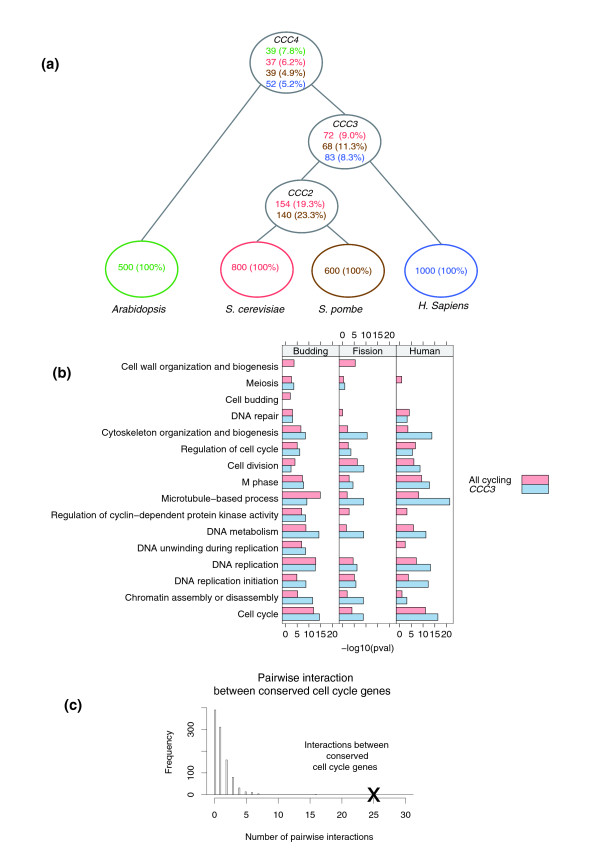
Conservation of cycling genes. **(a) **Percentage of conserved cycling genes in the four species. **(b) **Enrichment of cell cycle related Gene Ontology GO terms between all cycling genes and the CCC3 set in budding yeast, fission yeast, and humans. **(c) **Yeast protein-protein interactions [18]. We counted the number of interactions within a random set of 80 cycling yeast genes. In all, 1,000 sets were sampled. The histogram on the left plots the number of interactions observed for these sets. X represents internal interactions with the CCC3 set, which has significantly more internal interactions.

Figure 3a presents the number of conserved genes for the different evolutionary distances represented in our datasets. About 21% of the budding and fission yeast cycling genes reside in cliques containing genes from these two species (CCC2). When adding human genes, roughly 10% of cycling yeast genes and 8% of cycling human genes are included in such cliques (CCC3). Finally, between 5% and 7% of cycling genes in all four species are conserved in sequence and expression (CCC4). Additional data file 2 (Supporting Tables 4 to 10) presents the list of genes assigned to CCC4 and CCC3 for each of the species. We note that although our original sequence similarity criterion was based on BLAST e-values, following the clique analysis the resulting sets are in very good agreement with curated homology databases [17]. For example, 82% of budding yeast genes in CCC2 have a curated fission yeast homolog in CCC2. Similarly, 82% of fission yeast genes in CCC2 have a curated budding yeast homolog in CCC2. See our supporting website [15] for complete homology references.

To test the agreement of our conserved lists with complementary high-throughput datasets, we have repeated and extended our analysis discussed above but focusing only on genes included in CCC3 and CCC4. As Figures 2 and 3 and Additional data file 1 (Supporting Figure 4) show, CCC3 and CCC4 genes exhibit much stronger cell cycle characteristics when compared with the original set of cycling genes for each species. For example, in a protein-protein interaction dataset for budding yeast [18,19], genes in CCC3 are involved in ten times more pair-wise interactions when compared with a random set of similar size from the full set of cycling genes (Figure 3c). This indicates that these genes have long been involved in the same function. Similarly, the percentages of human genes bound by two cell cycle transcription factors are much higher for the CCC3 and CCC4 sets (Nrf1 and E2f2 [20]; Additional data file 1 [Supporting Figure 4d]). Also, for humans the CCC3 and CCC4 sets are much more repressed in several nonproliferating tissues when compared with the full set of cycling human genes (Figure 2c). Similarly, CCC4 genes exhibit stronger cell cycle characteristics in *Arabidopsis *and fission yeast expression experiments (Figure 2d-e).

We have also repeated our analysis by comparing our lists with subsets of cycling genes with high amplitude in each of the four species. As shown in Additional data file 1 (Supporting Figure 5), high amplitude genes exhibit similar cell cycle characteristics to the CCC3 and CCC4 sets for human and plants. However, for the two yeasts these high amplitude genes are more similar to the full set of cycling genes. This indicates that expression analysis alone cannot be used to identify this core set of genes.

### Motif analysis for budding and fission yeast genes

To further validate our findings of a large overlap between the cycling genes in the two yeast species, we turned to motif analysis. Several transcription factors are conserved between budding and fission yeast [21]. A possible explanation for expression conservation (or lack thereof) is in the conservation (or lack of conservation) of a binding motif for these cycling genes.

We started by looking at genes bound by the budding yeast factor Swi6, which regulates transcription at the G1/S transition [22]. We extracted three lists for this factor. The first, denoted BY6, contained cycling budding yeast genes in CCC2 determined to be bound by Swi6 [23]. The second list, denoted FY6C, contained fission yeast genes that both were in CCC2 and had homologs in BY6. These genes were determined to be cycling and conserved by our method. The third list (FY6NC) contained noncycling fission yeast genes with cycling budding yeast homologs bound by Swi6. This latter list serves as a negative control because it contains genes that have lost their cycling status between the two species. Four motif finders were run on each dataset; SOMBRERO [24,25], BioProspector [26], Consensus [27], and AlignACE [28] (see Materials and methods, below, for details). All four motif finding algorithms were able to identify the Swi6 motif in BY6 and FY6C, indicating that this motif is conserved between the two species, at least for some of the conserved cycling genes (Additional data file 1 [Supporting Figures 6 and 7]). In sharp contrast, none of these motif finders was able to identify the Swi6 motif in the upstream regions of genes in FY6NC.

### Mechanistic similarities and differences between cell cycle regulation in budding and fission yeast

We have extended the motif analysis discussed above to study ten additional transcription factors that were determined to play a key role in regulating cycling genes in budding yeast [3,16]. For each of these factors we extracted all cycling budding yeast genes determined to be bound by this factor [23] and their fission yeast homologs. As we did for Swi6, we further divided the fission yeast genes into two sets; the first contains fission yeast genes in CCC2 and the second (a negative control list) contains noncycling fission yeast homologs of cycling budding yeast genes. Next, we ran the four motif finders on each dataset.

The results are presented in Table 1 and Additional data file 1 (Supporting Figures 6 to 17). In Table 1 we report on the number of motif finders that identified the correct motif for each factor and on the percentage of genes in the set that contained this motif. Similar to the results obtained for Swi6, the other two G1/S factors, namely Swi4 and Mbp1, exhibit the optimal motif conservation pattern; the expected motifs are found in both the fission yeast cell cycle genes and the positive control of conserved budding yeast cell cycle genes, but are not found in the negative control set of noncycling fission yeast genes. Motif scan analysis (Additional data file 2 [Supporting Table 11]) confirms the results for these factors. For G2/M, the Fkh2 sets display similar, although less significant, pattern (two of four motif finders identified the correct motif for the cycling set). However, Fkh1 and Fkh2 motifs also appear, although less strongly, in the negative control sets. In total, FKH-like motifs are present in eight of the 11 negative control datasets. The M/G1 phase analysis is complicated by small dataset size. This may result from the lack of conservation between the two species for this phase [21]. As a result, motif match for this set is either weak (Swi5) or nonexistent (Mcm1 and Yox1).

**Table 1:** Summary of motif-finding results

Budding yeast phase	Transcription factor	Fission yeast cell cycle genes		Negative control (fission yeast non-cell-cycle genes)		Positive control (conserved budding yeast cell-cycle genes)		Extended positive control (all budding yeast CC genes)	
G1/S	Swi4	4	43%	0	0%	4	96%	4	98%
	Swi6	4	97%	0	0%	4	100%	4	83%
	Mbp1	4	59%	0	0%	4	93%	4	91%

G2/M	Fkh1	0	0%	2	22%	1	62%	3	67%
	Fkh2	2	45%	2	24%	1	74%	2	67%
	Ndd1	0	0%	0	0%	4	100%	4	100%

M/G1	Mcm1^a^	0	0%	0	0%	3	87%	4	88%
	Ace2	4^b^	86%	0	0%	0^b^	0%	4	88%
	Swi5	~2^b^	100%	0	0%	~2^b^	75%	1	0%
	Yox1	0^b^	0%	0^b^	0%	3^b^	86%	3	86%
	Yhp1	0^b^	0%	0^b^	0%	1^b^	0%	~1^b^	67%

Motif analysis of the conserved cycling genes in budding and fission yeast. For each set and each factor we list the number of motif finders (up to four) that identified the correct motif. Each motif finder often recovers multiple correct motifs, and each motif is associated with a list of predicted instances in promoter regions. We report the percentage of promoters that contain instances predicted by at least one-third of the correct motifs. The first and third columns are the CCC2 genes in budding and fission yeast, respectively. The second column is non-cycling fission yeast genes with homolog cycling budding yeast genes. See Additional data file 3 for further details. ^a^Mcm1 regulates genes in G2/M and M/G1. ^b^These datasets contain ten genes or fewer. ~, weak matches to the known motif.

### The biologic importance of the core set of cycling genes

**Figure 4:**
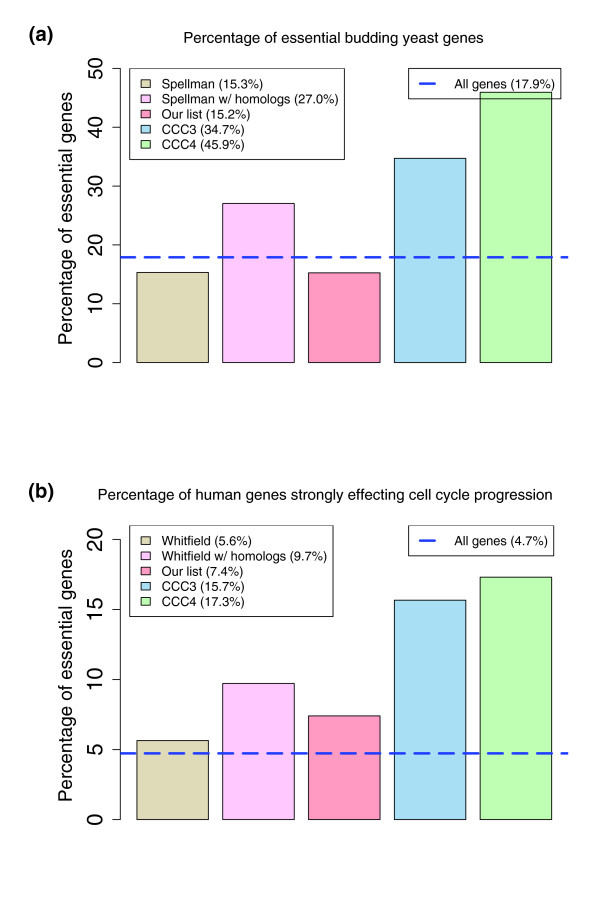
The importance of the core cycling genes. **(a) **Percentage of essential genes in different sets of budding yeast genes [29]. Although 18% of budding yeast genes are essential, only 15% of cycling genes are essential. Our analysis resolves this apparent contradiction by showing that the conserved cycling genes lists contain a much higher percentage of essential genes (35% and 46% for CCC3 and CCC4). Sequence alone cannot account for this high percentage (27%), indicating the importance of the combined analysis. **(b) **Similar analysis for the human lists using data from RNA interference knockdown experiments [30].

To further validate that genes in CCC3 and CCC4 are core cycling genes, we studied their importance using deletion data. Surprisingly, only 15% of cycling yeast genes are essential in rich media conditions [29], which is roughly equal to the overall percentage of essential yeast genes (18%). However, as Figure 4 shows, 35% of budding yeast genes in the CCC3 list and 46% of the genes in the CCC4 lists are essential. To test whether similar result could be obtained using only sequence data (without expression data for the other species), we extracted from the full list of cycling budding yeast genes those with homologs in all other species, without taking into account their cycling status in these other species. Although this increased the percentage of essential genes (to 27%), these percentages remained well below those achieved for CCC4, which uses the expression data.

We have also carried out similar analyses for human genes using data from RNA interference (RNAi) experiments [30]. In these experiments 24,373 genes were knocked down using RNAi and assessed for phenotypic influence on cell growth. For 1,152 (4.7%) of the genes, the resulting knockdown cells presented phenotypic growth defects. As Mukherji and coworkers [30] note in their report, roughly 6% of cycling human genes reported by Whitfield and colleagues [1] are included in this list. Similar to the process we conducted in yeast, we considered sequence data only and extracted from the Whitfield list those genes with homologs in the other three species. For this list, the percentage of genes increases to 10%. Again, the most enriched lists are obtained when using the CCC3 and CCC4 sets. For these, the percentage climbs to 16% (CCC3) and 17% (CCC4). These findings highlight the importance of the conserved set and support our conclusion that it contains key cycling genes.

### Conserved protein complexes regulated by the cell cycle

To determine cell cycle regulated protein complexes conserved between these species, we searched for protein complexes with one or more subunits in the CCC3 set using high-throughput protein-protein interaction data. This type of data is thus far only available in budding yeast [18,19]. Additional data file 2 (Supporting Table 12) and Additional data file 1 (Supporting Figure 18) present some of the protein complexes that we identified. Some of these complexes are known to regulate important events in the cell cycle. For example, the origin recognition complex (ORC) is a well conserved complex that is involved in the initiation of DNA synthesis [31]. Other examples are the cohesin complex, which is responsible for binding the sister chromatids during mitosis after S phase [32], and the ribonucleoside-diphosphate reductase (RNR) complex, which is involved in the maintenance of the cellular pool of dNTPs [33].

### Gene Ontology analysis of conserved cycling genes

The CCC3 list gives us our first look at the conserved core of periodically transcribed genes across evolution. Even though CCC3 contains relatively few genes (0.4% to 1.3% of the total number of genes for each species), many of these genes play a role in key processes required for growth. Using Gene Ontology (GO) analysis [34], we identified categories that were enriched in this set. For budding yeast these categories include cell cycle (*P *= 3 × 10^-15^), DNA replication (*P *= 2 × 10^-13^), and mitosis (*P *= 1 × 10^-7^). Similar enrichments were found for human conserved cycling genes and for fission yeast. For example, cell cycle (*P *= 5 × 10^-17^), DNA replication (*P *= 7 × 10^-14^) and cell division (*P *= 2 × 10^-9^) are enriched in humans, and cell cycle (*P *= 10^-9^) and chromatin assembly/disassembly (*P *= 10^-9^) are enriched in fission yeast. Figure 3 and Additional data file 2 (Supporting Tables 13 to 21) present *P *values for the various GO categories.

Some categories were more enriched in the CCC3 set than in the full list. For these categories, CCC3 contains a disproportionate number of genes when compared with the overall percentage of cycling genes. This indicates that many of the genes associated with these functions have been conserved in cyclic expression between the species. These include categories related to DNA metabolism (*P *= 5.7 × 10^-12 ^for CCC3 and *P *= 1.1 × 10^-6 ^for the full list) and chromatin assembly (*P *= 3.7 × 10^-5 ^versus *P *= 0.01). In contrast, there are a number of categories that are much more enriched in the full list, indicating that they have probably evolved, or at least greatly expanded, in the individual species. These include categories such as mitosis for fission yeast (*P *= 1.6 × 10^-4 ^versus *P *= 3.6 × 10^-7^) and the cell wall category, which exhibits a great deal of species-specific variation between the budding yeast, the fission yeast, and metazoans [35]. For the human list, DNA repair and chromosome segregation were more significantly enriched in the full set (*P *= 5.9 × 10^-4 ^versus *P *= 7.3 × 10^-5^, and *P *> 0.1 versus *P *= 9.0 × 10^-8^, respectively). Although these functions are conserved across organisms, our analysis indicates that many of these genes are cycling only in human cells, perhaps indicating that these functions have been adapted to accommodate the longer cell cycle.

### Analysis of specific CCC3 genes

Partial functional knowledge is available for all but one (YPL247C) of the 72 budding yeast genes on CCC3. Sixteen of these genes encode products that are involved in DNA replication and another 23 are involved in chromosome organization and biogenesis. These include structural components (Mcms, tubulins, and histones) as well as regulatory proteins (cyclins, Cdc20, and Cin8). The *mcm2 *(*cdc19*) and *mcm6 *genes were previously known to be cyclic subunits of the highly conserved Mcm pre-replication complex in fission yeast [5,36]. Our combined analysis indicates that two other genes (*mcm3 *and *mcm5*) may also be periodic, similar to the budding yeast and human Mcm subunits. Another large class of conserved cyclic genes is involved in chromosome segregation (*ASE1*, *KIP1*, *NUM1*, and *STU2*) and cytokinesis (*MOB1*, *HOF1*, *KEL2*, and *IQG1*). In addition, the list includes factors that affect transcription globally (*ARP7 *and *TUP1*) and specifically (*ACE2*, *FKH2*, and *HCM1*). Interestingly, the S phase specific transcription factor Hcm1 has a conserved cyclic transcript, as do 22 of its predicted targets [3]. The fact that nearly 30% of the budding yeast CCC3 genes are potential targets of Hcm1 is consistent with the known role of Hcm1 in regulating genes involved in chromosome dynamics [3,37].

There is only a small number of genes in CCC3 that are not obviously involved in cell cycle specific processes. These genes include three involved in metal homeostasis (*SMF2*, *SMF3*, and *CTH2*), some cell wall proteins (*FIG2*, *AGA1*, and *SED1*) and alkaline phosphatase (*PHO8*). These gene products could be involved in unknown aspects of the cell division cycle, or they could be evolutionarily related to other cell cycle proteins.

## Conclusion

By applying a combined analysis, coupled with an unbiased homology metric, we were able to identify a large set of genes as conserved in sequence and cycling status between four different species: budding and fission yeast, human, and *Arabidopsis*.

A number of previous efforts to compare cycling gene lists derived independently for each species concluded that only a small number of genes are conserved between these species. For example, Rustici and coworkers [4] concluded that only 5% to 10% of cycling budding yeast genes have a cycling homolog in fission yeast. Jensen and colleagues [10] identified only five orthologous groups as conserved between the four species (about 1% of the cycling genes) and only eight groups (2%) between the three species of CCC3. The differences between these conclusions can be attributed to differences in the analysis of the expression and sequence data, as mentioned in the Introduction (above). We note, however, that the results presented by Oliva and coworkers [5] provide partial support to our conclusions. Although they did not carry out a complete conservation analysis, they found that 72 of their top 200 cycling fission yeast genes (36%) had a cycling homolog in budding yeast. Earlier work that used clustering methods to look at global expression similarities between species also supports our findings regarding the extent of expression conservation [38,39]. Although our analysis identifies a larger fraction of conserved cycling transcripts than does that conducted by Jensen and colleagues [10], we find the same striking co-occurrence of cell cycle specific phosphorylation of the gene products they encode. As Additional data file 2 (Supporting Table 22) shows, when using data on Cdk1 phosphorylation [40] we find that 65% of tested CCC3 gene products are phosphorylated by Cdk1. This percentage is twice the percentage of phosphorylated gene products from the full set of tested cycling genes (33%) and eight times higher than the percentage of tested random genes (8%). These finding reinforces the view that there is a conserved core of genes that are regulated at multiple levels during the cell cycle in most eukaryotic cells.

Our results are strongly supported by the fact that genes conserved in two or more species display much stronger cell cycle characteristics than the full list for each species. They also show extensive interactions within the set, and almost half of the CCC4 yeast genes are essential. These observations and GO analysis indicates that these genes are crucial components of the cell cycle system. Combined, these findings support our claim that the lists we derive contain a core conserved set of cycling genes.

Our findings indicate that combined analysis of expression and sequence data leads to refined lists containing a core set of system specific genes. Although we have focused here on the cell cycle, such an analysis can be carried out to study a number of other biologic systems that have been profiled using expression experiments in multiple species, including immune response and circadian rhythm.

## Materials and methods

### Assigning cyclic status to genes

We applied a probabilistic graphical model to combine microarray expression data and sequence data for identification of cycling genes, as described in Lu and coworkers [12]. We used microarray expression data reported by Spellman [2], Rustici [4], Oliva [5], Peng [6], Whitfield [1], and Menges [7] and their coworkers. We downloaded protein sequences from the National Center for Biotechnology Information website [41]. The method starts by using gene specific expression data to compute a cycling score based on both the amplitude and periodicity [13]. We run BLASTALL [42] to calculate bit scores between all pairs of sequences, as was done by Sharan and coworkers [43]. We use a Markov random field to model the joint likelihood of the data. The (hidden) cycling status of each gene is represented by a node in the graph, and two nodes are connected by an edge if the bit score for the two genes is above a threshold. We define potential functions on nodes to capture information from the cycling scores, where we assume the scores of cycling and the noncycling genes follow a mixture of extreme value distributions, and define potential functions on edges to capture the correlation of cycling statuses between similar genes. The posterior beliefs of the cycling status of the genes are estimated using loopy belief propagation algorithm. Finally, we rank the genes by their posterior and use the name number as were used in the original papers (500 for *Arabidopsis*, 800 for budding yeast, 600 for fission yeast genes, and 1,000 human genes). See Additional data file 3 for complete details.

### Identifying conserved sets

Genes identified as cycling in each species were used to identify conserved sets of cycling genes. This is done using the Markov clustering algorithm (MCL) [14] as follows. First, we start with the graph of all cycling genes. Edges in the graph are defined based on the bit score cut-off, as mentioned above. Second, for any connected subgraph in this graph, we use MCL to break it into smaller subgraphs if it has more than 30 nodes. Third, repeat the previous step until all connected subgraphs have at most 30 nodes.

Next, we assign genes to different conserved sets based on the other species represented in the subgraph to which they belong. The numbers of genes in the conserved sets are shown in Figure 3, in which the sets are organized as a tree reflecting the evolutionary relation between the four species.

### Motif discovery

For each gene in the lists, the appropriate intergenic region was extracted from the budding or fission yeast genome. Four motif finders were run on each dataset: SOMBRERO [24,25], Consensus [27], BioProspector [26], and AlignACE [28]. Both SOMBRERO and BioProspector require a background model, and the background was constructed from all intergenic regions (in the appropriate genome) for both cases. SOMBRERO was run using default settings, and simultaneously using all known yeast motifs as an appropriate source of prior knowledge. Consensus and BioProspector were run using default settings requiring the top ten motifs to be reported. AlignACE was run using default settings (using a seed motif length of 10), and provided with the background intergenic GC content (31.45% for fission and 35.3% for budding). See Additional data file 3 for further details.

### *P *value analysis

GO enrichment *P *values were computed using STEM [44], which relies on hyper-geometric distribution. Corrected *P *values were computed by permutation analysis using STEM.

## Additional data files

The following additional data are available with the online version of this manuscript. Additional data file 1 provides supporting figures. Additional data file 2 provides supporting tables. Additional data file 3 provides further details regarding the methods used and results generated.

## Supplementary Material

Additional data file 1 Provided are supporting figures. Click here for file

Additional data file 2 Provided are supporting tables. Click here for file

Additional data file 3 Provided are supporting methodologic details. Click here for file
