# Supplementary material for: Combined analysis reveals a core set of cycling genes
Source: Genome Biol. 2007 Jul 24;8(7):R146. doi: 10.1186/gb-2007-8-7-r146 (PMC2323241; doi:10.1186/gb-2007-8-7-r146)
Supplement: Additional data file 1 — Provided are supporting figures. [file gb-2007-8-7-r146-S1.pdf]

Supporting Figures for:

Combined analysis reveals a core set of cycling genes

Yong Lu, Shaun Mahony, Panayiotis V. Benos, Roni Rosenfeld, Itamar Simon, Linda L. Breeden, Ziv Bar-Joseph

Figures		Pages
Supporting figures 1-3:	Cliques of conserved cycling genes	2-4
Supporting figure 4:	Gene expression analysis for conserved cell cycle genes	5-6
Supporting figure 5:	Analysis of high amplitude genes	7-8
Supporting figures 6-17:	Motif analysis of conserved sets in yeast	9-20
Supporting figure 18:	Budding yeast complexes enriched with Conserved cycling genes	21
Supporting Figure 19:	Comparison of CCC2 with Peng *et al*	24

Supporting Figure 1. The advantage of combined analysis

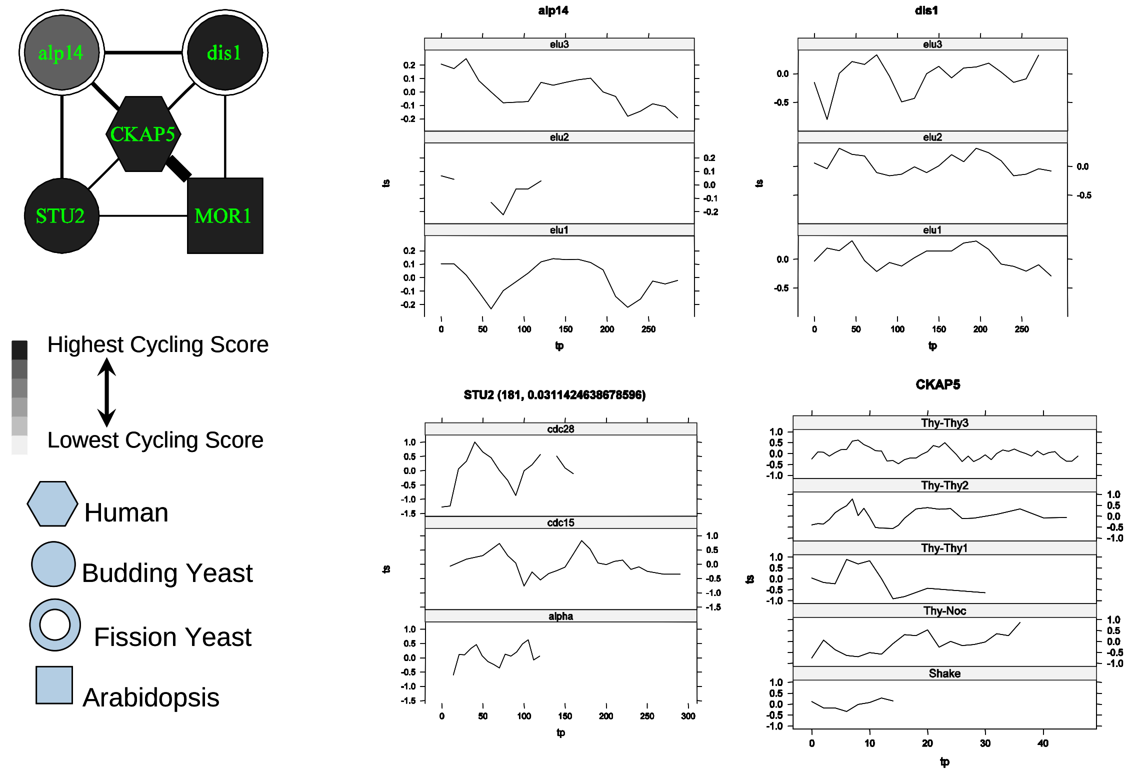

Supporting Figure 1. The advantage of combined analysis. The figure presents one of the cliques identified by our algorithm as consisting of cycling genes from all species. The color of the nodes corresponds to their cycling score (the darker, the higher the score, see Figure 1). Using the graph connectivity genes with cycling scores just below the cutoff can be elevated by considering their homlogs' scores. The genes in this clique encode TOG-related proteins, a family of microtubule-associated proteins (MAPs). Microtubules are part of the cytoskeletal systems in all eukaryotes, and their dynamics and organization are cell cycle regulated. Proteins in this group localize to the plus-end tips of microtubules and are essential for spindle pole organization. [48,54,55]. Alp14, a fission yeast gene, does not obtain a high cycling score due to missing data and is not included on the original list by Rustici *et al.* [4]. Using our method it was determined to be cycling due to its relatively high score and cycling homologs.

Supporting Figure 2. The advantage of combined analysis

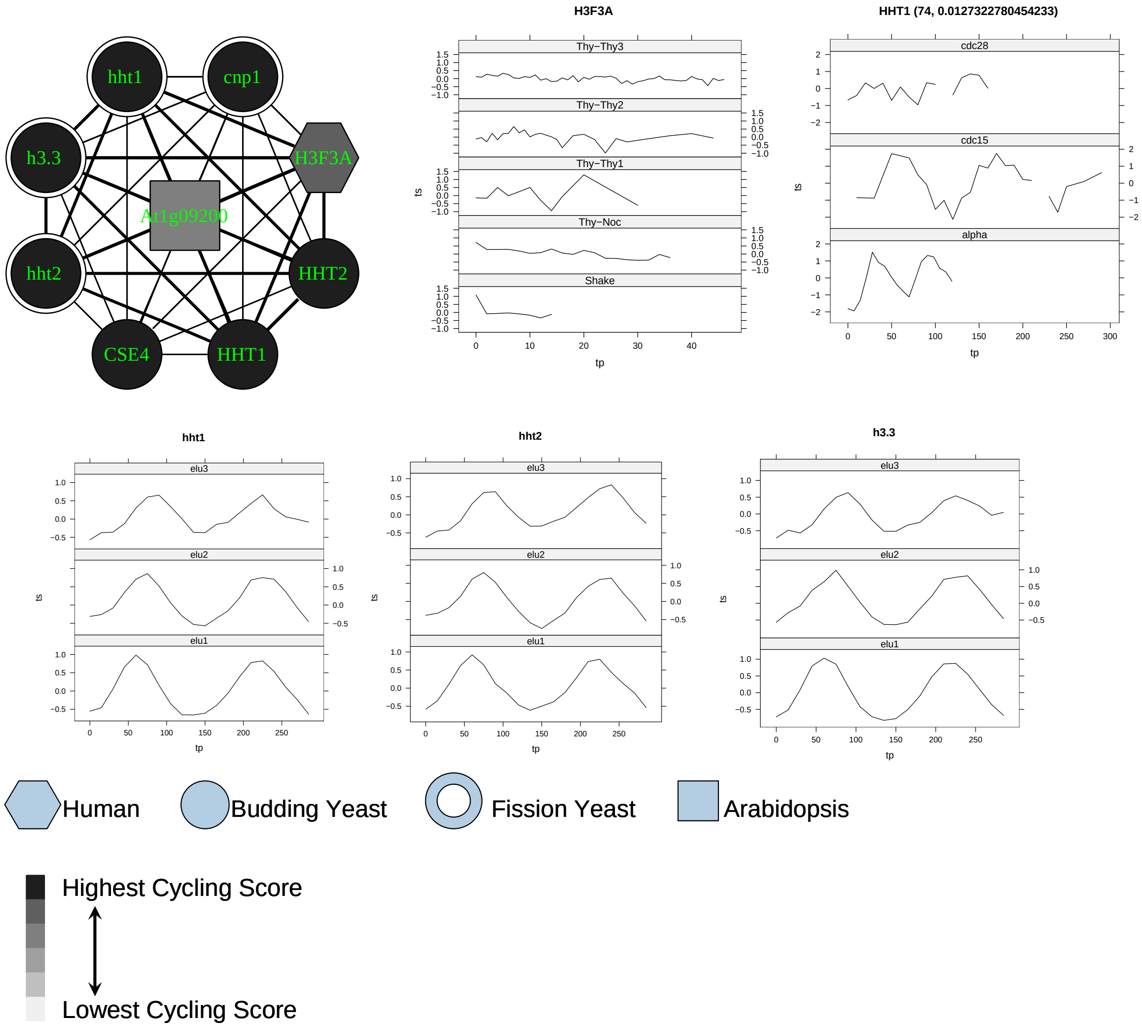

Supporting Figure 2. Similar analysis for Histone H3 and variants. Histones are proteins responsible for the nucleosome structure of the chromosomal fiber in eukaryotes. Cse4 is a centromere protein similar to H3, and is required for proper chromatid segregation [56]. Cnp1 is a H3 variant also required for chromatid segregation [57].

Supporting Figure 3. The advantage of combined analysis

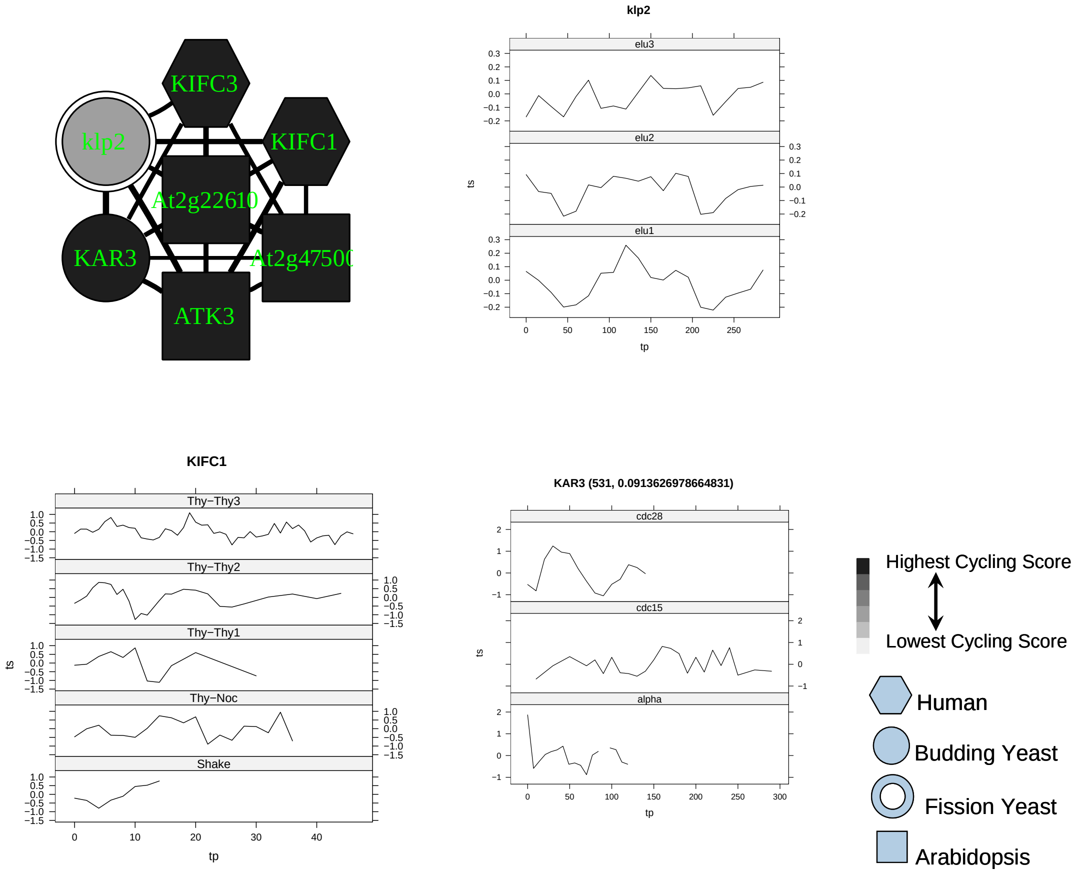

Supporting Figure 3. Genes in this group are kinesin-like proteins. Fission yeast Klp2 promotes spindle disassembly [58]. Budding yeast Kar3 is required for syster chromatid cohesion [59]. Human Kifc1 is believed to be necessary for proper cytokinesis [60]. Klp2 is not included in the original list by Rustici *et al.*

Supporting Figure 4. Complementary high throughput analysis

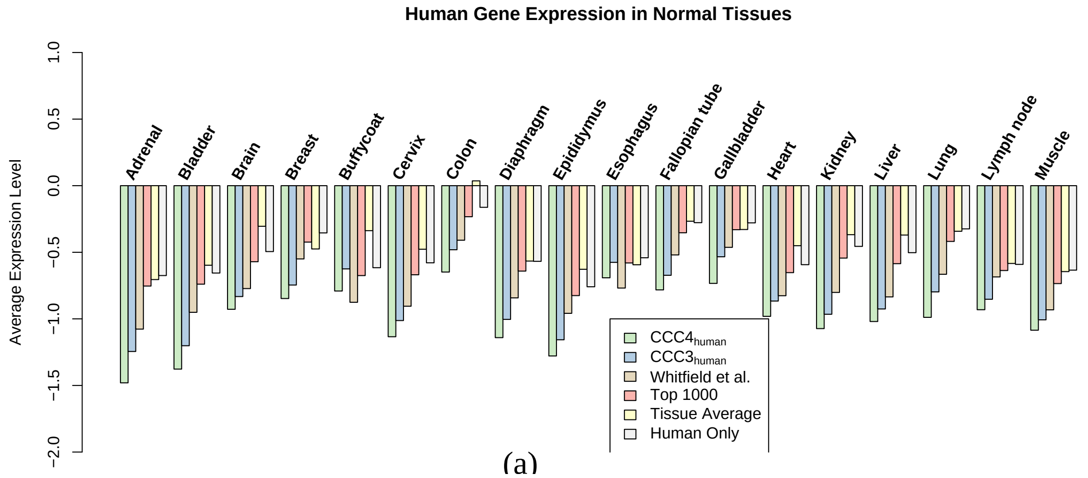

(a)

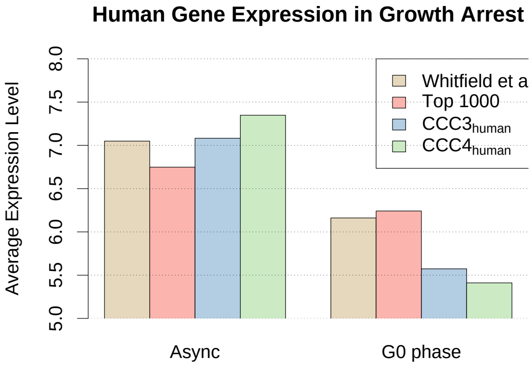

(b)

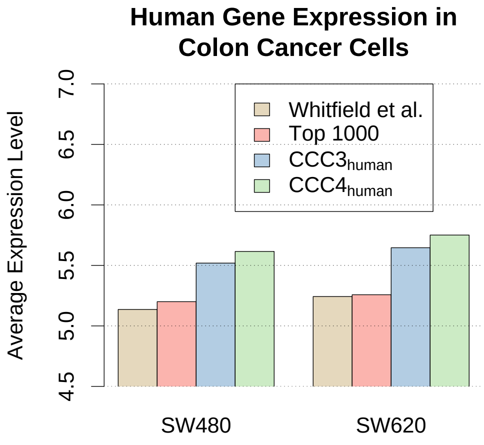

(c)

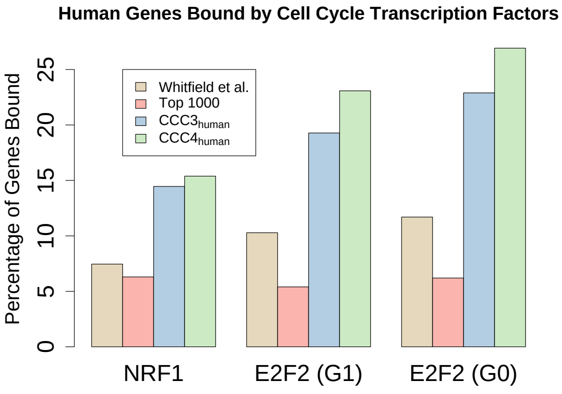

(d)

(e)

Supporting Figure 4. Additional high throughput analysis. (a): expression levels of human genes in normal tissues, using data from Shyamsundar *et al.* [51]. Genes in the conserved set have lower expression levels for most non-proliferating normal tissues when compared to the full list and the list of Whitfield *et al.* [1]. (b): Human gene expression level in asynchronous cells and G0 cells. The expression of the whole cycling list is lower in G0 phase than in asynchronous population. The change of the conserved set of cycling genes is the most drastic among all sets compared. (Significance of difference from all cycling genes: p-value for CCC3 = 0.005, p-value for CCC4 = 0.006). (c) Human gene expression levels in colon cancer cells [61]. In both cases, our list and the original list have the same average expression values. The conserved sets have higher expression levels than both these lists (CCC3: p-value = 10^-5 and 2.6*10^-5, CCC4: p-value=0.006 and 0.009). (d): Percentage of human cycling genes bound by two cell cycle transcription factors. Data was retrieved from Ren *et al.* [20]. The CCC3 and CCC4 set have much higher percentage than the whole list.

Supporting Figure 5. Analysis of high amplitude genes

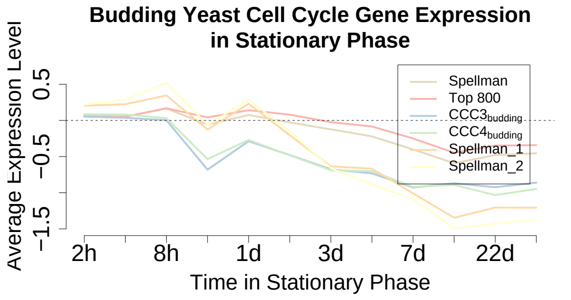

(a)

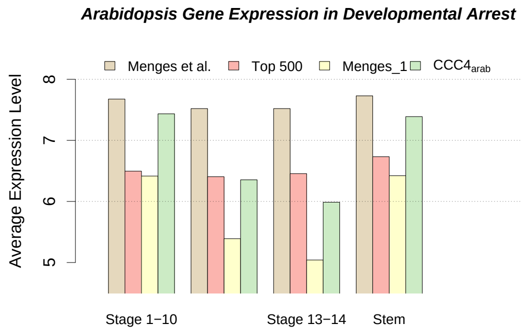

(b)

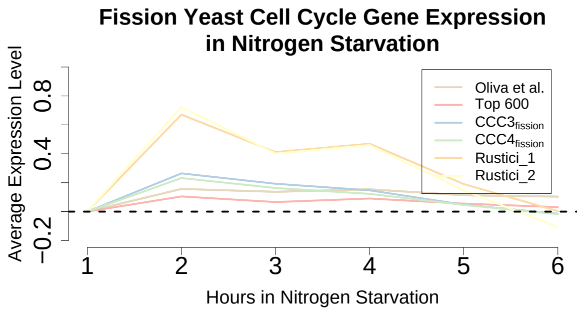

(c)

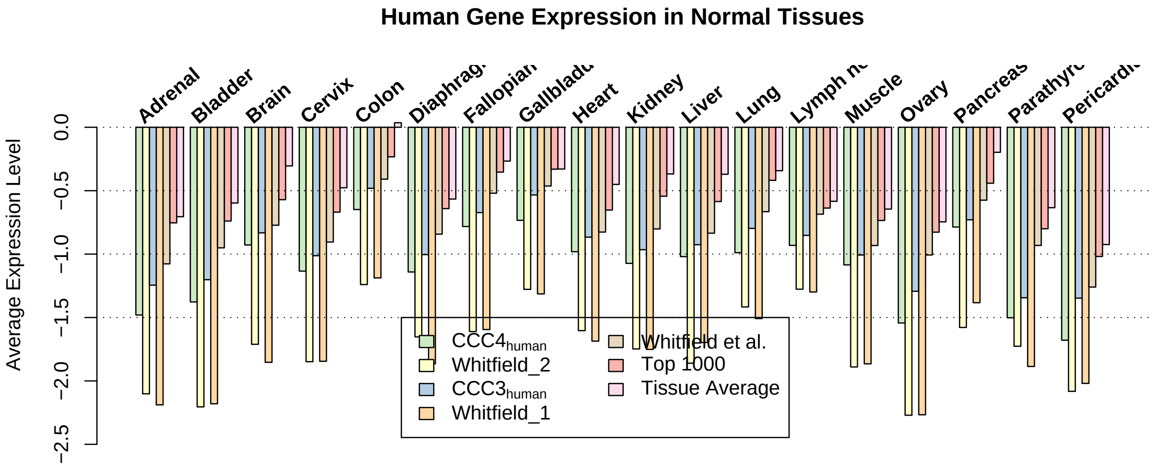

(d)

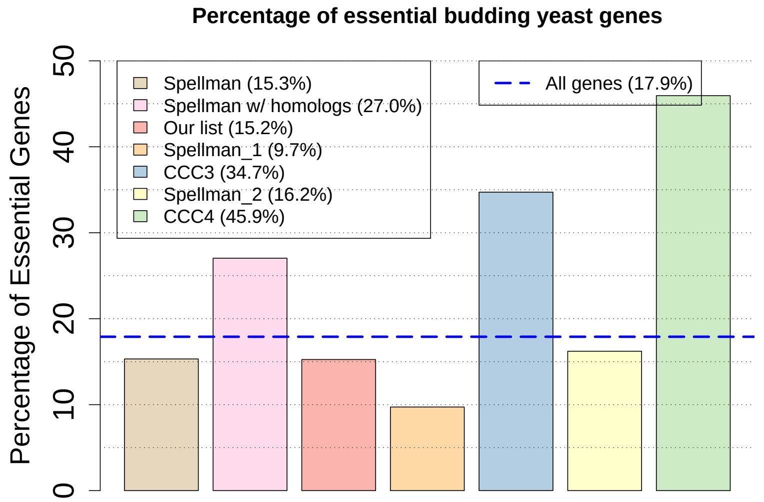

(e)

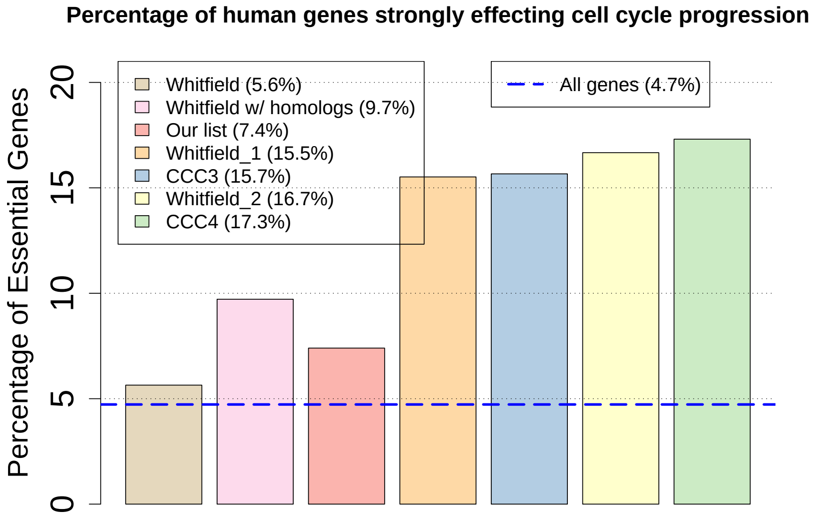

(f)

Supporting Figure 5. High throughput analysis with an additional control sets. These plots are similar to those in Fig 2 (b-e) and Fig 4 of the main text, with two additional control sets. For each species we selected the highest amplitude genes in the original lists [1,2,4,7], with the same size as CCC3 and CCC4 (only CCC4 for the plant data). While these sets show strong characteristic of cycling genes (similar to the conserved sets) in the human and plant datasets, they are similar to the general cycling genes in the yeasts. For example, essentiality analysis for yeast (e) indicates that the percentage of essential high amplitude genes is similar to the percentage of essential genes in the full list of cycling genes and is much lower than the percentage of essential genes in CCC3 and CCC4. However, for human genes (f) the percentage of essential genes within the high amplitude set is higher than the percentages of essential genes in the full list though it is still slightly lower than the percentages in CCC3 and CCC4. Similar results can be seen using complementary expression analysis (a-d).

<u>Supporting Figure 6: *S. cerevisiae* Cell Cycle SWI6 (conserved genes)</u>

The SWI6 dataset contains 31288 bases in 25 sequences (average: 1251.52 bp).

SWI6's binding motif as found by Harbison *et al.* is shown below:

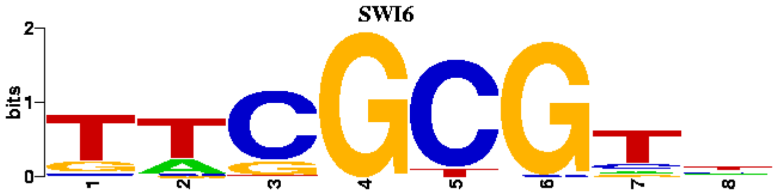

SOMBRERO (ranks 1-10), Consensus (ranks 1-10), BioProspector (ranks 1-4, 7, 9 & 10) and AlignACE (rank 2) all recover a motif similar to the above.

S. cerevisiae **CC Cons SWI6 SOMBRERO top 10 motifs**

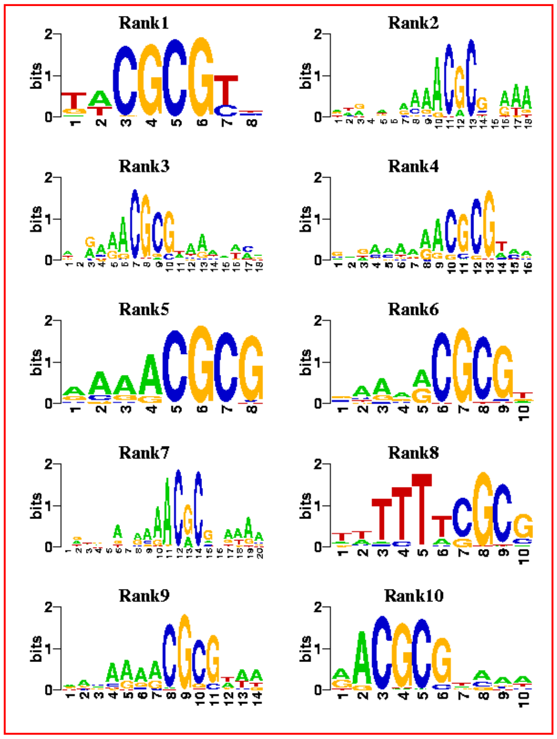

S. cerevisiae **CC Cons SWI6 BioProspector top 10 motifs**

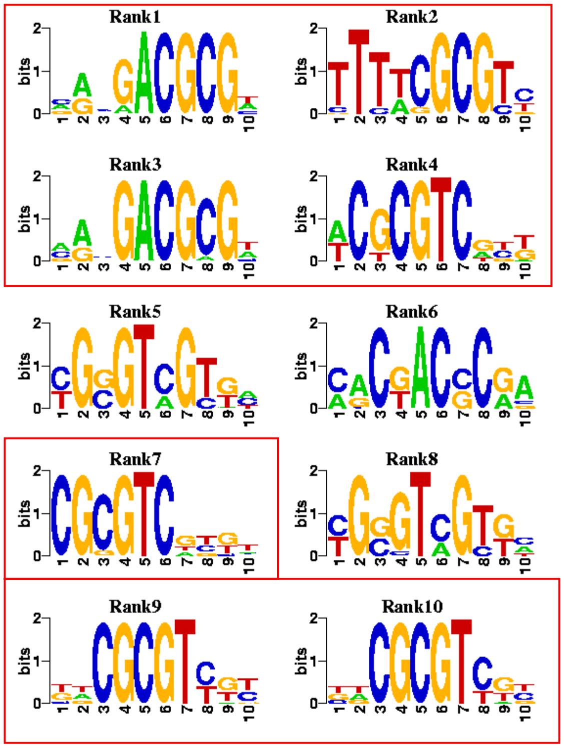

Supporting Figure 7: *S. pombe* Cell Cycle SWI6 (conserved genes)

The SWI6 dataset contains 56735 bases in 35 sequences (average: 1621.00 bp).

SWI6's binding motif as found by Harbison *et al.* is shown below:

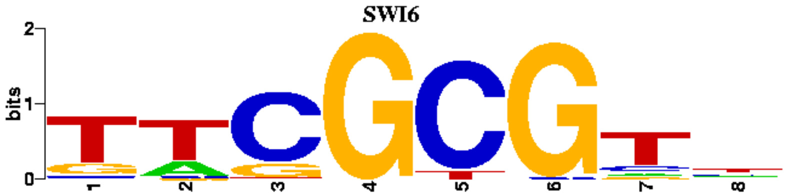

The above motif is found by SOMBRERO (ranks 2-6), Consensus (ranks 1-10), BioProspector (ranks 1-10), and AlignACE (rank 6). Secondary motifs found in the data include a FKH-like motif (SOMBRERO & AlignACE).

<u>*S. pombe* CC Cons SWI6 SOMBRERO top 10 motifs</u>

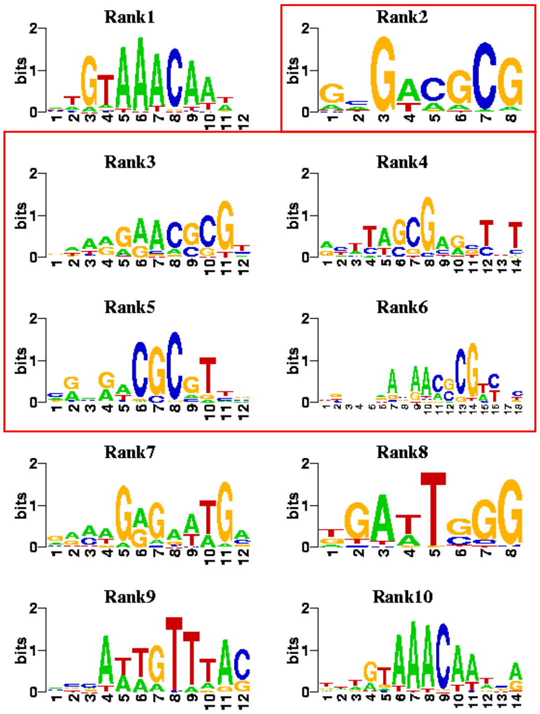

<u>*S. pombe* CC Cons SWI6 Consensus top 10 motifs</u>

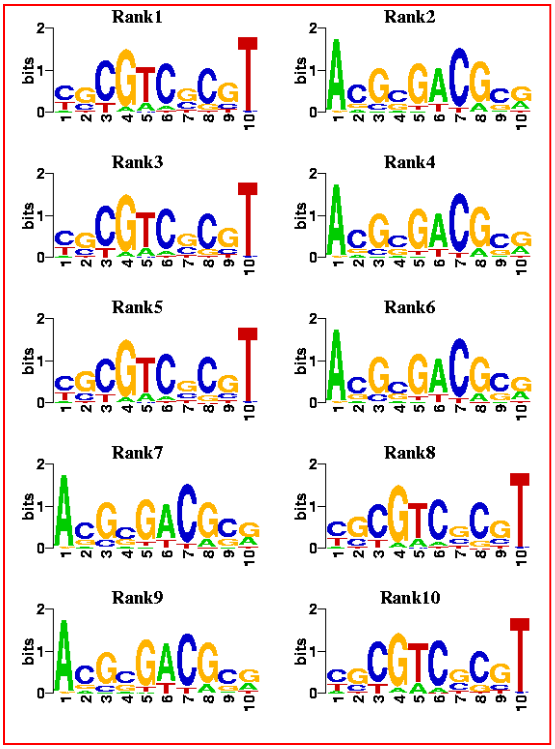

Supporting Figure 8: *S. cerevisiae* Cell Cycle SWI4 (conserved genes)

The SWI4 dataset contains 46536 bases in 28 sequences (average: 1662.00 bp).

SWI4's binding motif as found by Harbison *et al.* is shown below:

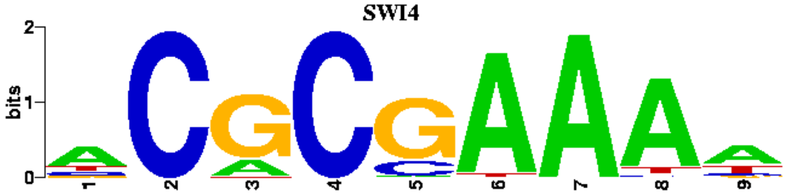

SOMBRERO (ranks 1, 4, 6), Consensus (ranks 1-10), BioProspector (ranks 2-5, 7-10) and AlignACE (rank 9) all recover a motif similar to the above. SOMBRERO also recovers a "GGAAT" pattern motif (ranks 2, 3, 8, 10).

***S. cerevisiae* CC Cons SWI4 SOMBRERO top 10 motifs**

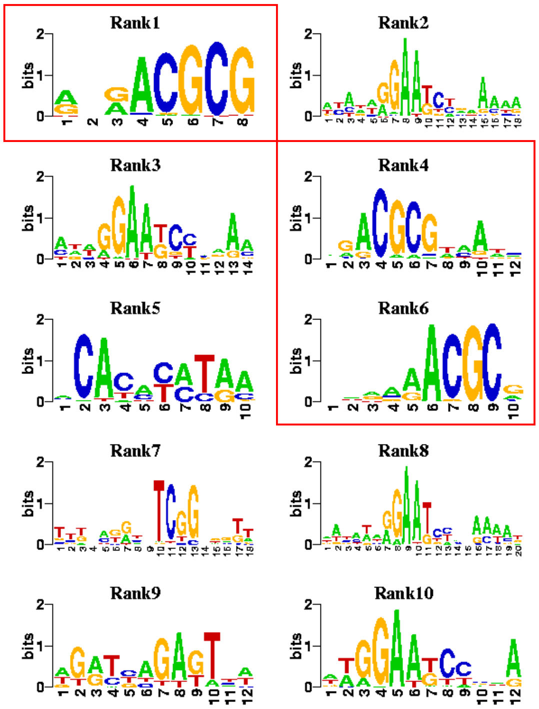

***S. cerevisiae* CC Cons SWI4 Consensus top 10 motifs**

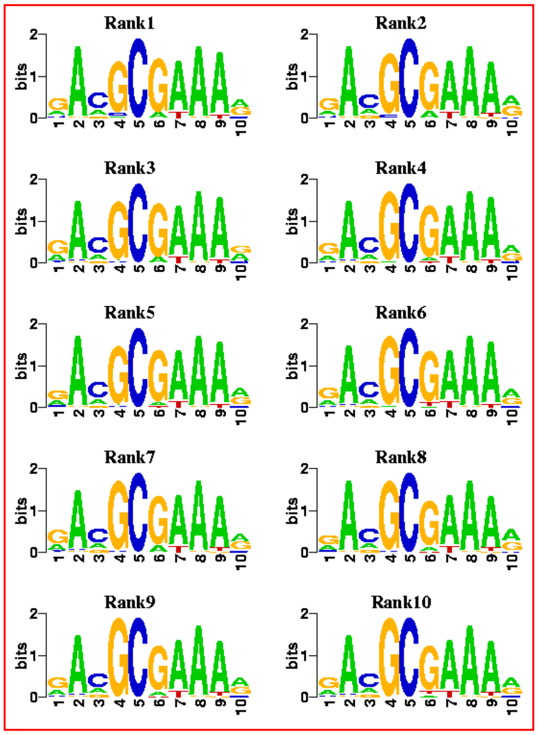

Supporting Figure 9: *S. pombe* Cell Cycle SWI4 (conserved genes)

The SWI4 dataset contains 55552 bases in 37 sequences (average: 1501.41 bp).

SWI4's binding motif as found by Harbison *et al.* is shown below:

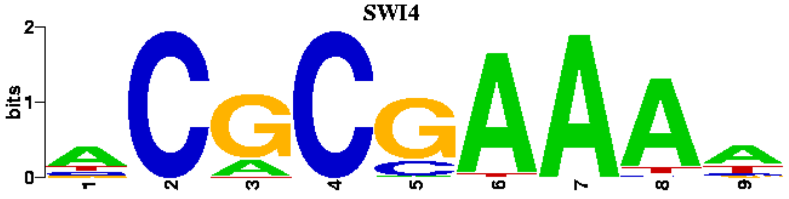

The above motif is found by SOMBRERO (ranks 1-5), Consensus (rank 10), BioProspector (ranks 1-6, 8, & 10), and AlignACE (rank 2). Secondary motifs found in the data include an FKH-like motif (SOMBRERO, BioProspector & AlignACE) and an ACE2-like motif (SOMBRERO, Consensus & BioProspector).

S. pombe CC Cons SWI4 SOMBRERO top 10 motifs

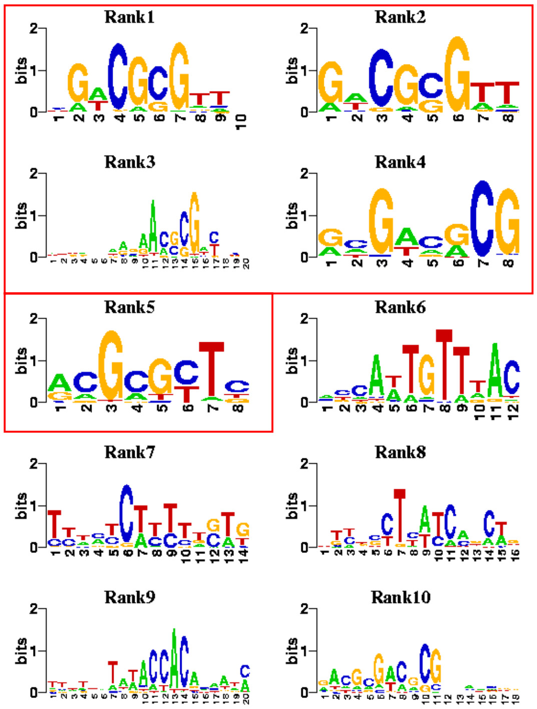

S. pombe CC Cons SWI4 BioProspector top 10 motifs

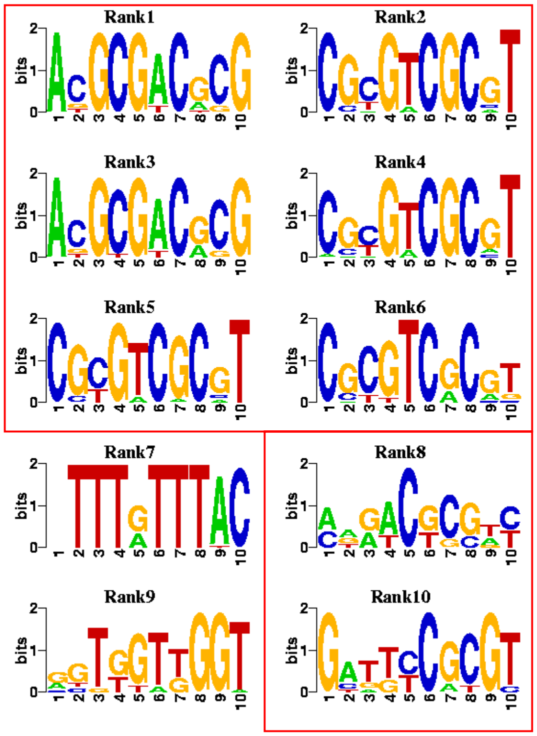

<u>Supporting Figure 10: *S. cerevisiae* Cell Cycle MBP1 (conserved genes)</u>

The MBP1 dataset contains 30826 bases in 29 sequences (average: 1062.97 bp).

MBP1's binding motif as found by Harbison *et al.* is shown below:

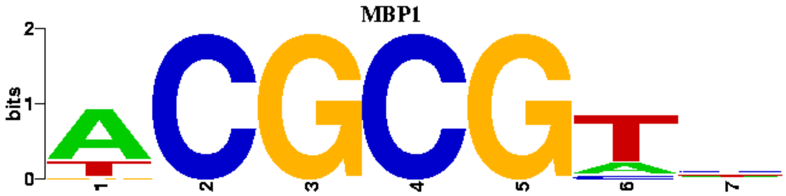

SOMBRERO (ranks 1-10), Consensus (ranks 1-10), BioProspector (ranks 1-10) and AlignACE (rank 2) all recover a motif similar to the above.

<u>*S. cerevisiae* CC Cons MBP1 SOMBRERO top 10 motifs</u>

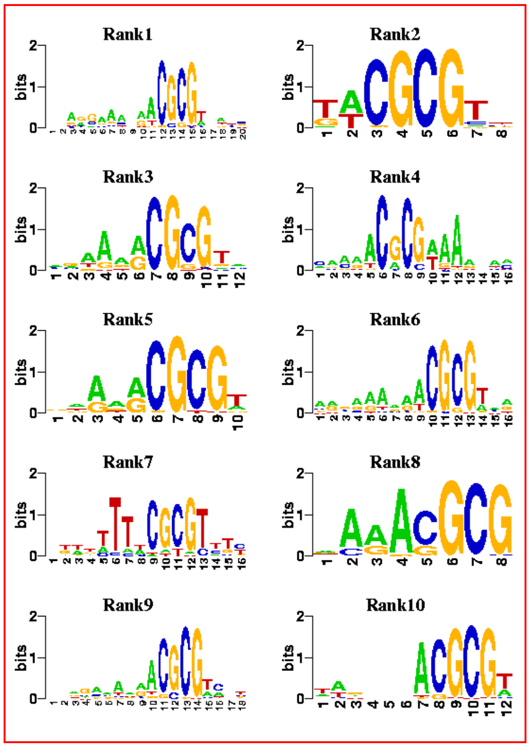

<u>*S. cerevisiae* CC Cons MBP1 BioProspector top 10 motifs</u>

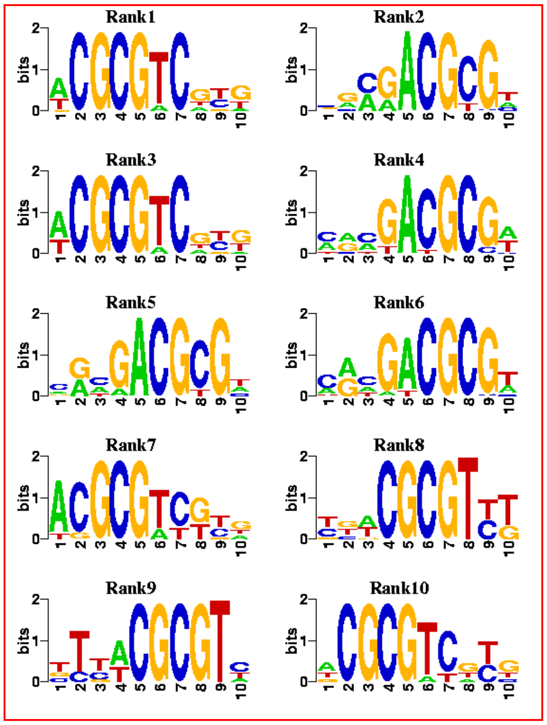

Supporting Figure 11: *S. pombe* Cell Cycle MBP1 (conserved genes)

The MBP1 dataset contains 59916 bases in 44 sequences (average: 1361.73 bp).

MBP1's binding motif as found by Harbison *et al.* is shown below:

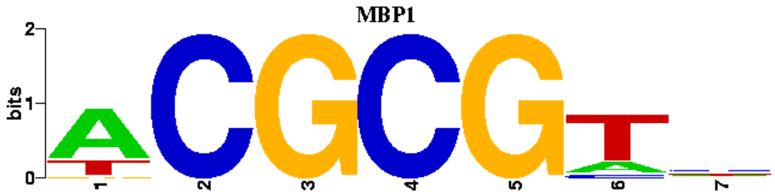

The above motif is found by SOMBRERO (ranks 1-10), Consensus (ranks 1-10), BioProspector (ranks 1-10), and AlignACE (rank 2). Secondary motifs found in the data include an FKH-like motif (AlignACE) and a "TGTTG" motif (SOMBRERO).

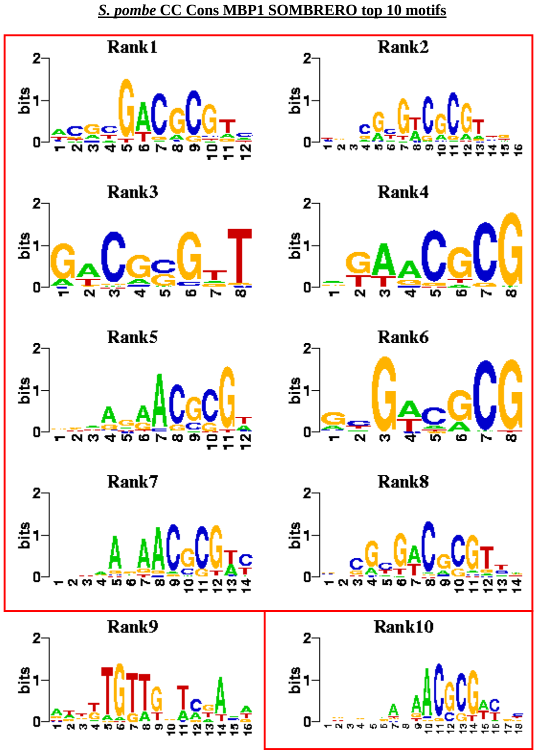

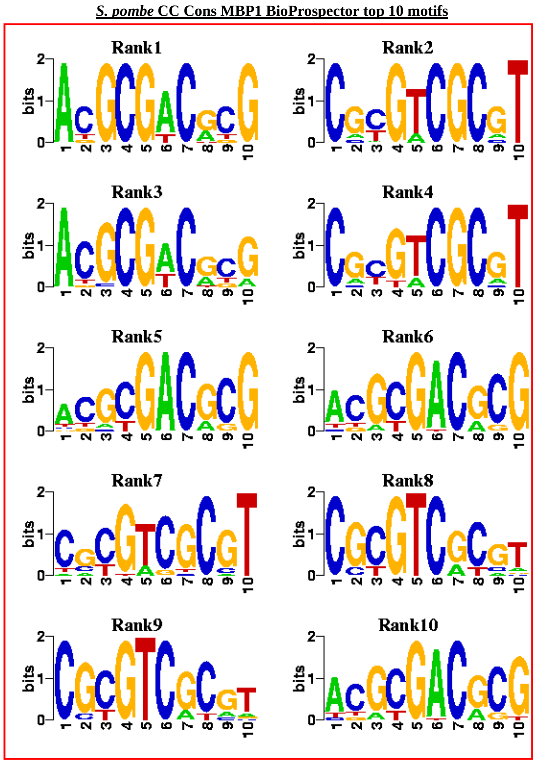

Supporting Figure 12: *S. cerevisiae* Cell Cycle FKH2 (conserved genes)

The FKH2 dataset contains 17241 bases in 23 sequences (average: 749.61 bp).

FKH2's binding motif as found by Harbison *et al.* is shown below:

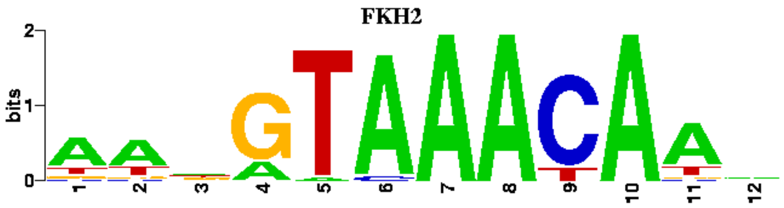

Only BioProspector (ranks 1, 3-5) recovers a motif similar to the above. Both SOMBRERO and AlignACE recover MCM1-like motifs and BioProspector finds a "AGGGT"-containing motif.

S. cerevisiae CC Cons FKH2 BioProspector top 10 motifs

S. cerevisiae CC Cons FKH2 SOMBRERO top 10 motifs

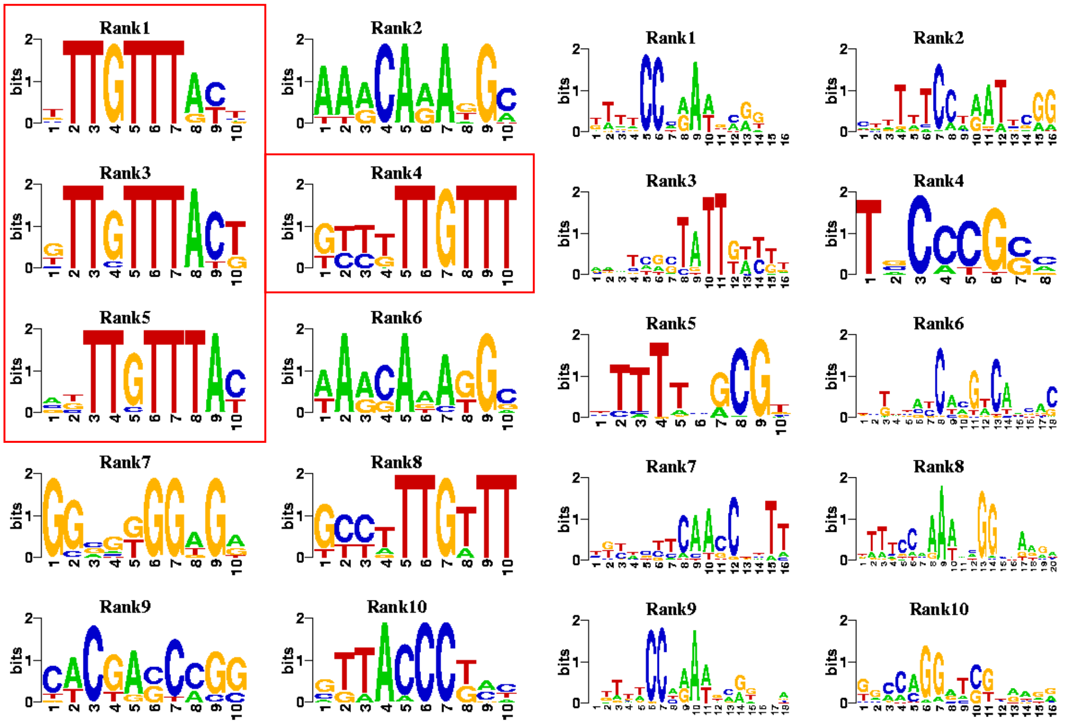

<u>Supporting Figure 13: *S. pombe* Cell Cycle FKH2 (conserved genes)</u>

The FKH2 dataset contains 54392 bases in 40 sequences (average: 1359.80 bp).

FKH2's binding motif as found by Harbison *et al.* is shown below:

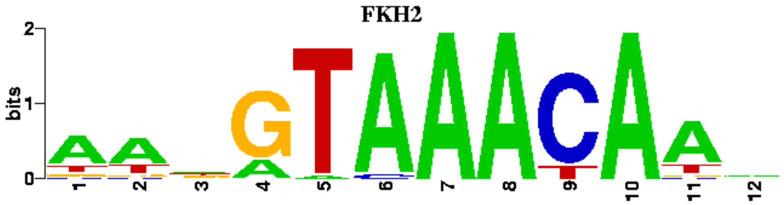

The above motif is found by SOMBRERO (rank 2) and AlignACE (rank 3). Secondary motifs found in the data include an SWI4/6-like motif (BioProspector), a "GACGC" motif (Consensus & BioProspector) and a "AGGGT" motif (BioProspector & AlignACE).

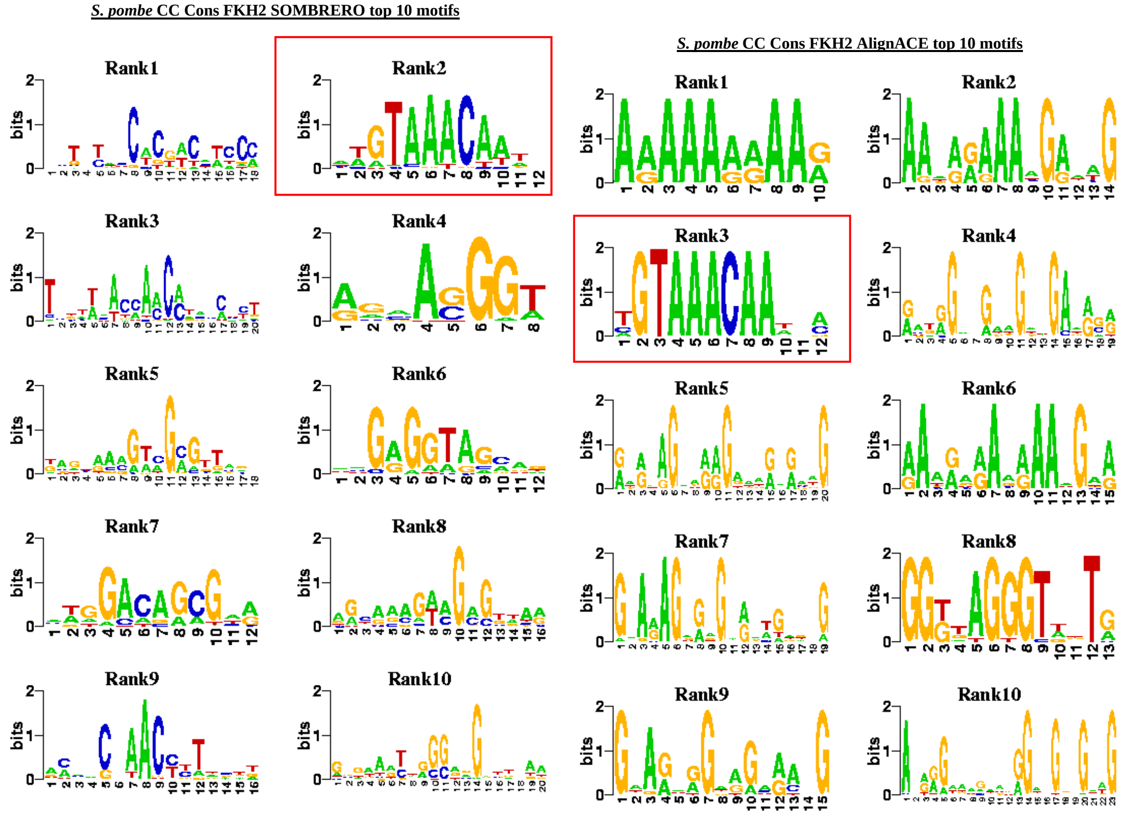

Supporting Figure 14: *S. cerevisiae* Cell Cycle ACE2 (conserved genes)

The ACE2 dataset contains 1583 bases in 3 sequences (average: 527.67 bp).

ACE2's binding motif as found by Harbison *et al.* is shown below:

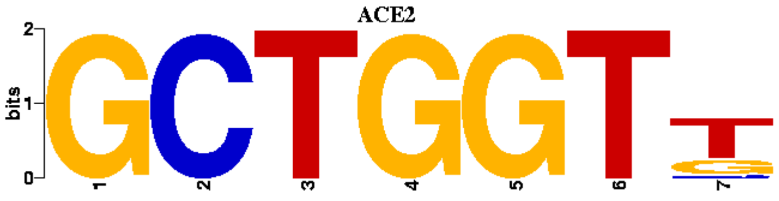

None of the motif-finders recover motifs similar to the above. Consensus (rank 9) recovers a SWI4/6-like motif.

S. cerevisiae CC Cons ACE2 SOMBRERO top 10 motifs

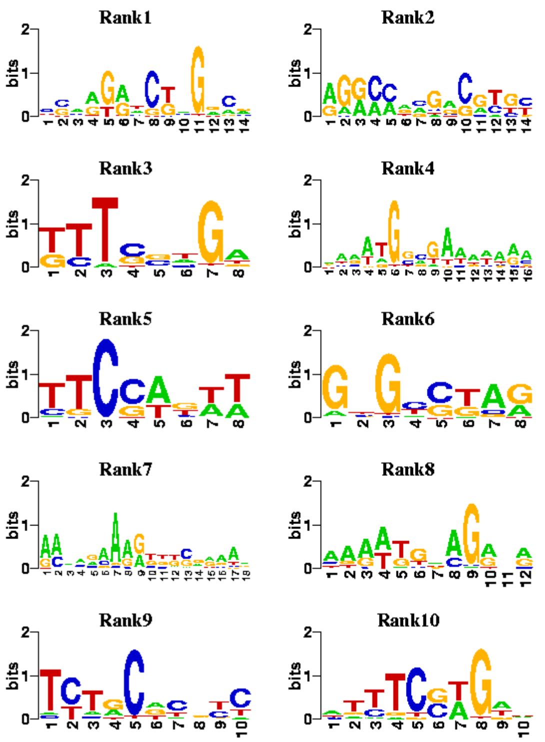

S. cerevisiae CC Cons ACE2 Consensus top 10 motifs

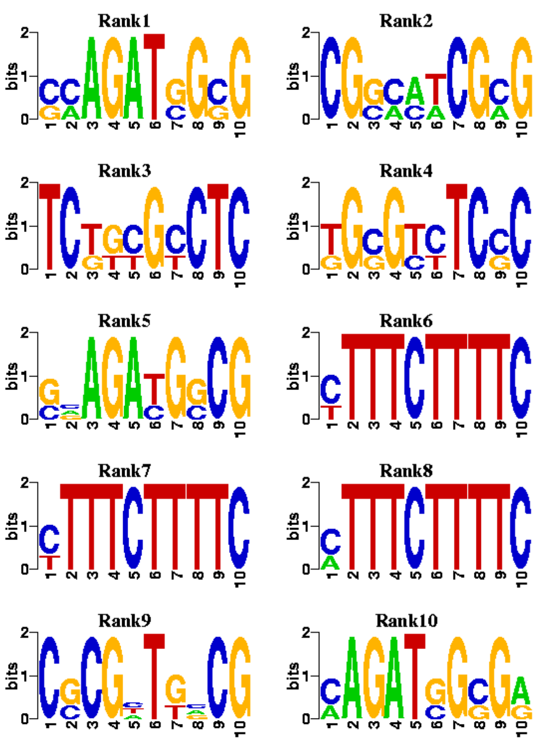

<u>Supporting Figure 15: *S. pombe* Cell Cycle ACE2 (conserved genes)</u>

The ACE2 dataset contains 8810 bases in 7 sequences (average: 1258.57 bp).

ACE2's binding motif as found by Harbison *et al.* is shown below:

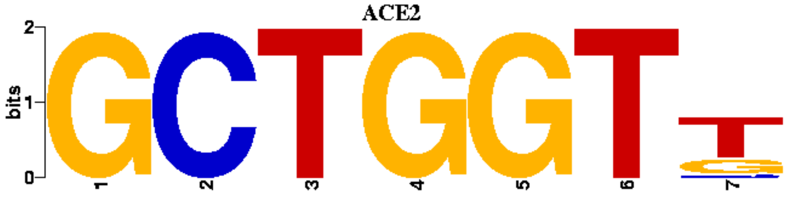

All motif-finders recover a partial match to the above motif (motifs containing the "TGGT" pattern); SOMBRERO (ranks 1-3), Consensus (ranks 1-5 & 7), BioProspector (ranks 1-4, 7-10), and AlignACE (rank 9).

<u>*S. pombe* CC Cons ACE2 SOMBRERO top 10 motifs</u>

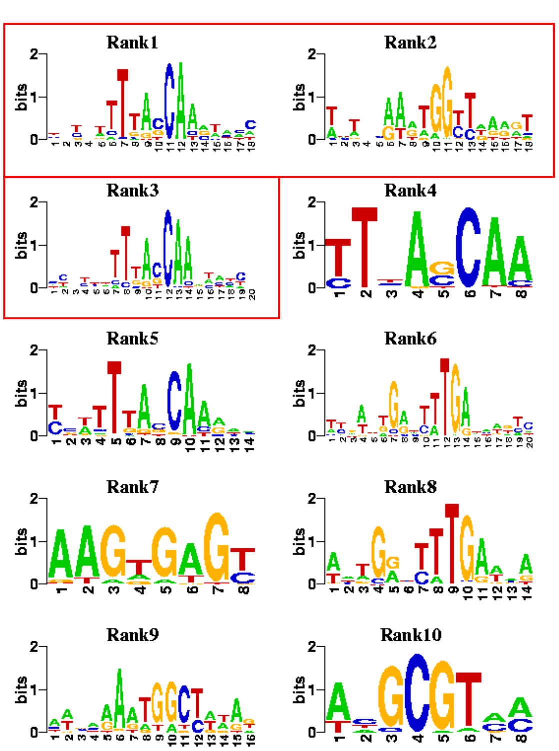

<u>*S. pombe* CC Cons ACE2 BioProspector top 10 motifs</u>

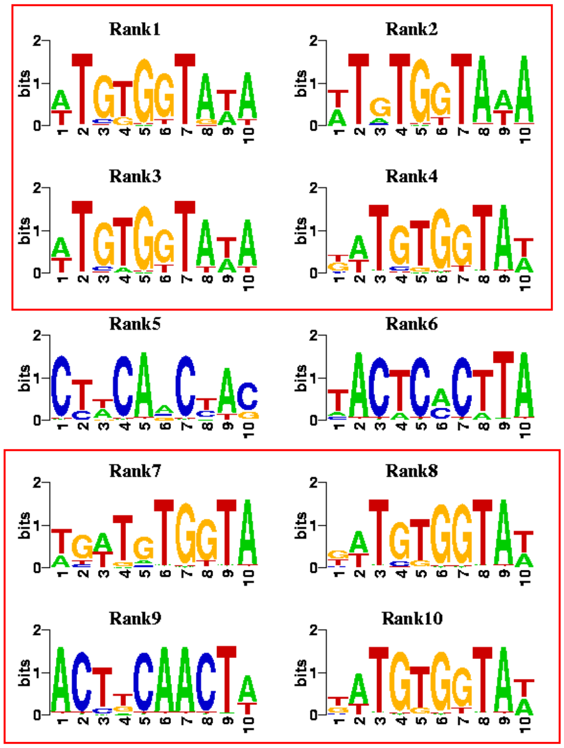

Supporting Figure 16: *S. cerevisiae* Cell Cycle SWI5 (conserved genes)

The SWI5 dataset contains 2539 bases in 4 sequences (average: 634.75 bp).

SWI5's binding motif as found by Harbison *et al.* is shown below:

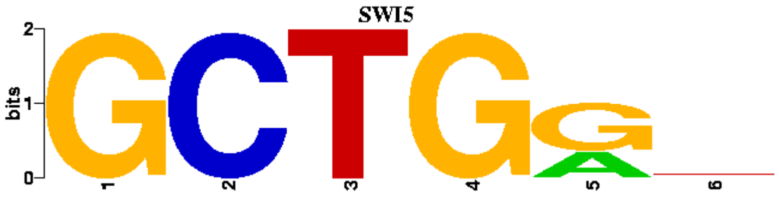

SOMBRERO (rank 6) and Consensus (rank 4) both find weakly similar motifs to the above (i.e. "GCTG"-containing motifs). Note that this dataset has limited sequence data.

S. cerevisiae CC Cons SWI5 SOMBRERO top 10 motifs

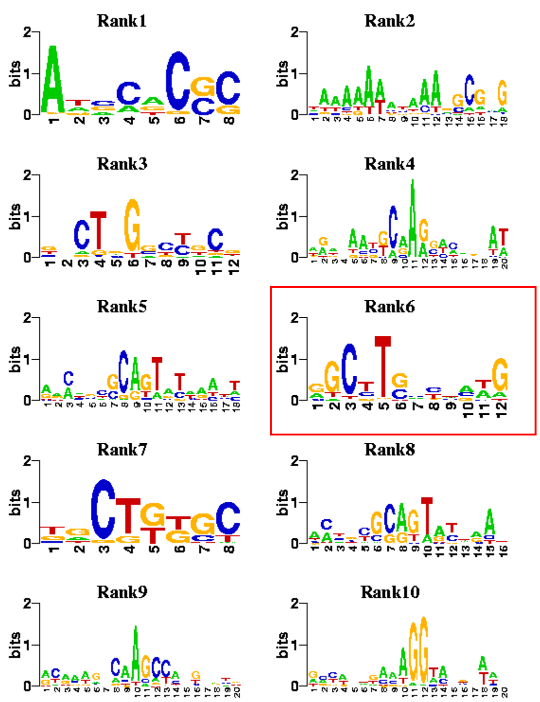

S. cerevisiae CC Cons SWI5 Consensus top 10 motifs

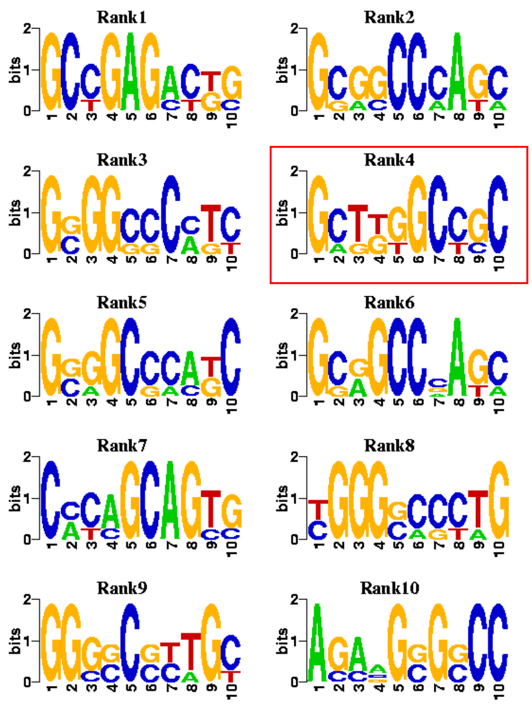

Supporting Figure 17: *S. pombe* Cell Cycle SWI5 (conserved genes)

The SWI5 dataset contains 4844 bases in 4 sequences (average: 1211.00 bp).

SWI5's binding motif as found by Harbison *et al.* is shown below:

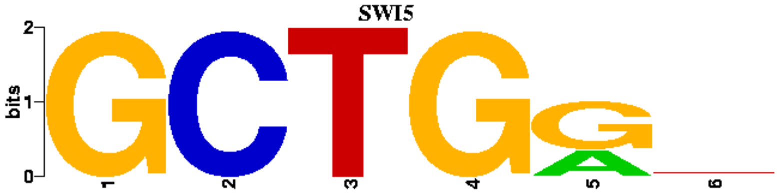

SOMBRERO (rank 7) and Consensus (ranks 1-3 & 6-10) recover partial matches to the above motif (motifs containing the "GCTG" core). SOMBRERO also recovers a FKH-like motif.

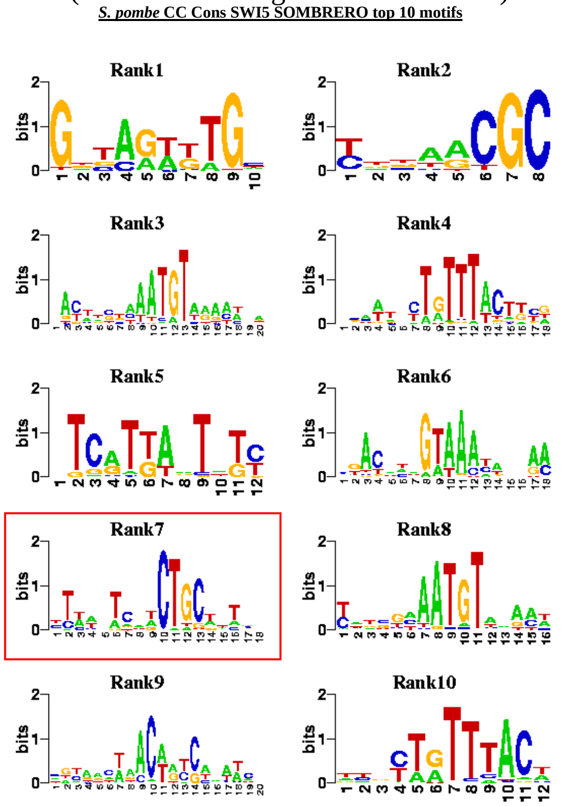

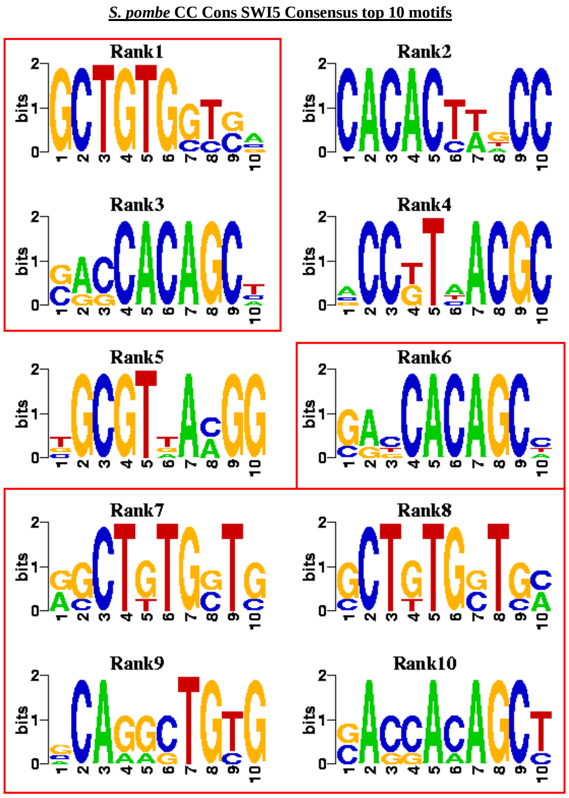

Supporting Figure 18. Budding yeast complexes

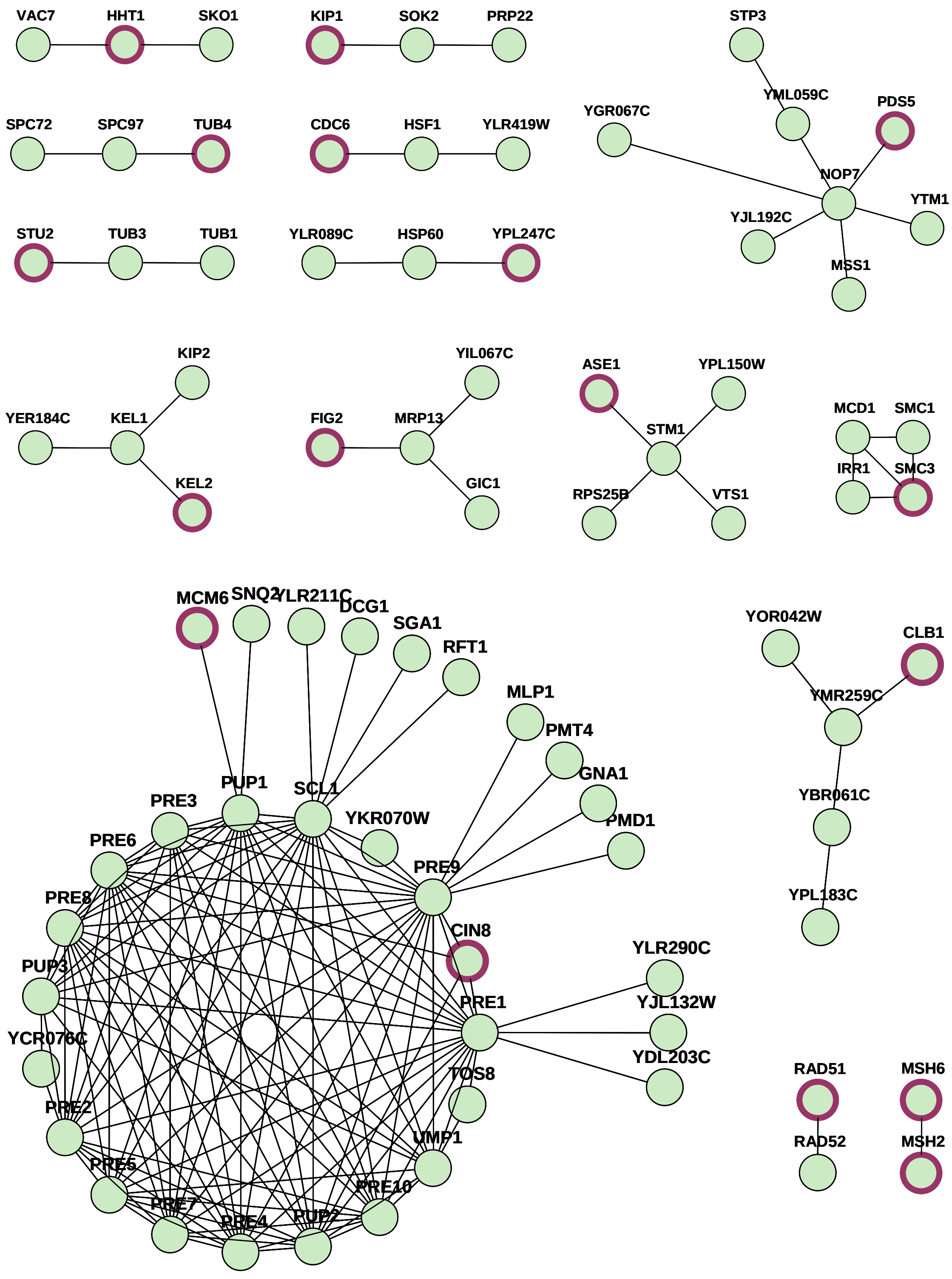

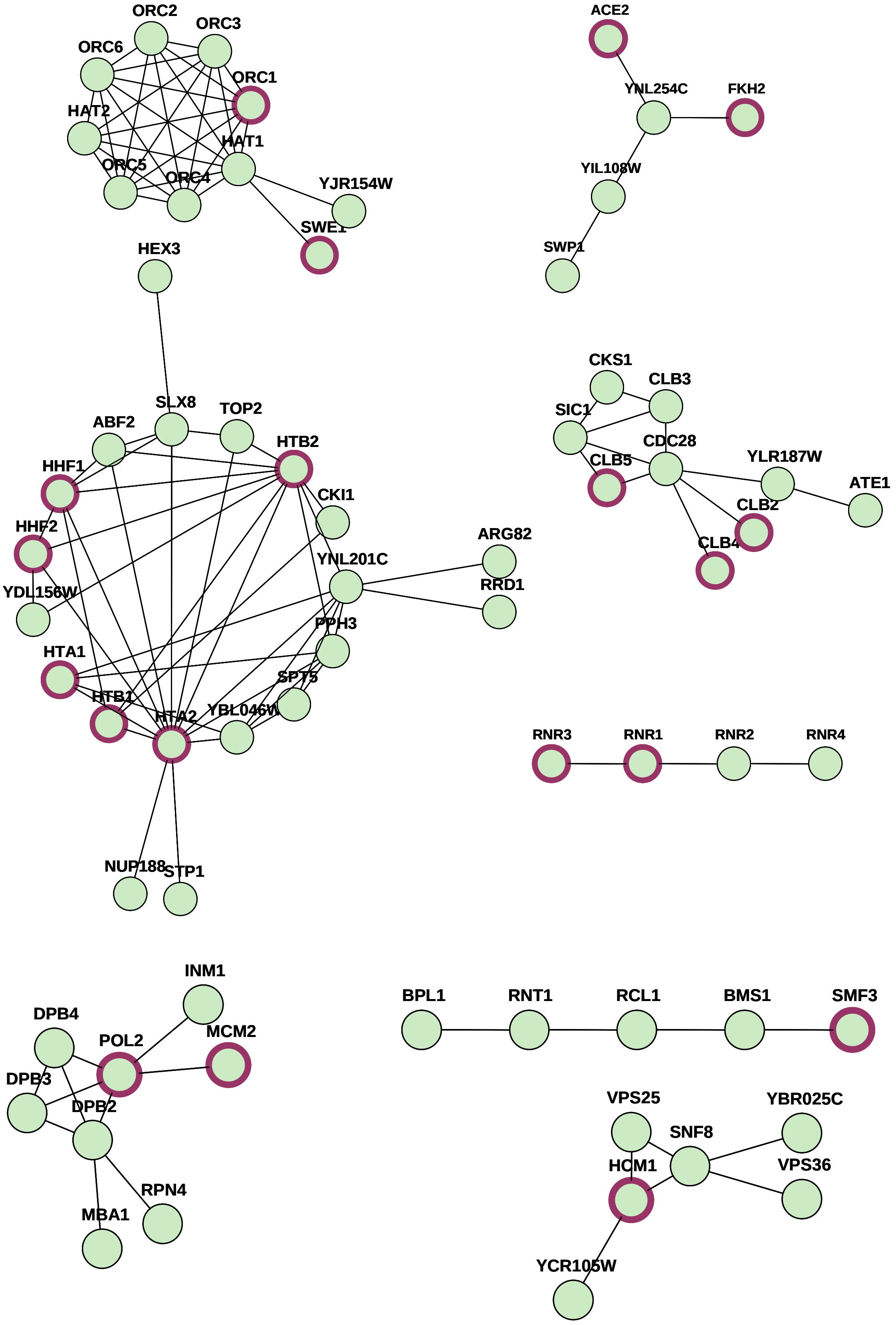
ORC2
ORC3
ORC6
ORC1
HAT2
HAT1
ORC5
ORC4
YJR154W
SWE1
HEX3
ACE2
YNL254C
FKH2
YIL108W
SWP1
CKS1
CLB3
SIC1
CDC28
CLB5
YLR187W
ATE1
CLB2
CLB4
SLX8
TOP2
ABF2
HTB2
HHF1
CKI1
HHF2
ARG82
YNL201C
RRD1
YDL156W
PPH3
HTA1
SPT5
HTB1
HTA2
YBL046W
RNR3
RNR1
RNR2
RNR4
NUP188
STP1
INM1
DPB4
MCM2
POL2
DPB3
DPB2
RPN4
MBA1
BPL1
RNT1
RCL1
BMS1
SMF3
VPS25
YBR025C
SNF8
HCM1
VPS36
YCR105W

Supporting Figure 18. Enrichment for conserved genes in budding yeast complexes. We present complexes containing genes in the CCC3 set, using data from Krogan *et al.* [18]. The cycling subunits are highlighted in purple. Some of these complexes are known to regulate important events in the cell cycle. For example, the origin recognition complex (ORC) is a well conserved complex involved in the initiation of DNA synthesis [62]. Another example is the cohesin complex, which is responsible for binding the sister chromatids during mitosis after S phase [32]. See supporting complex tables for details and enrichment analysis.

Supporting Figure 19. Comparison of CCC2 with Peng *et al*

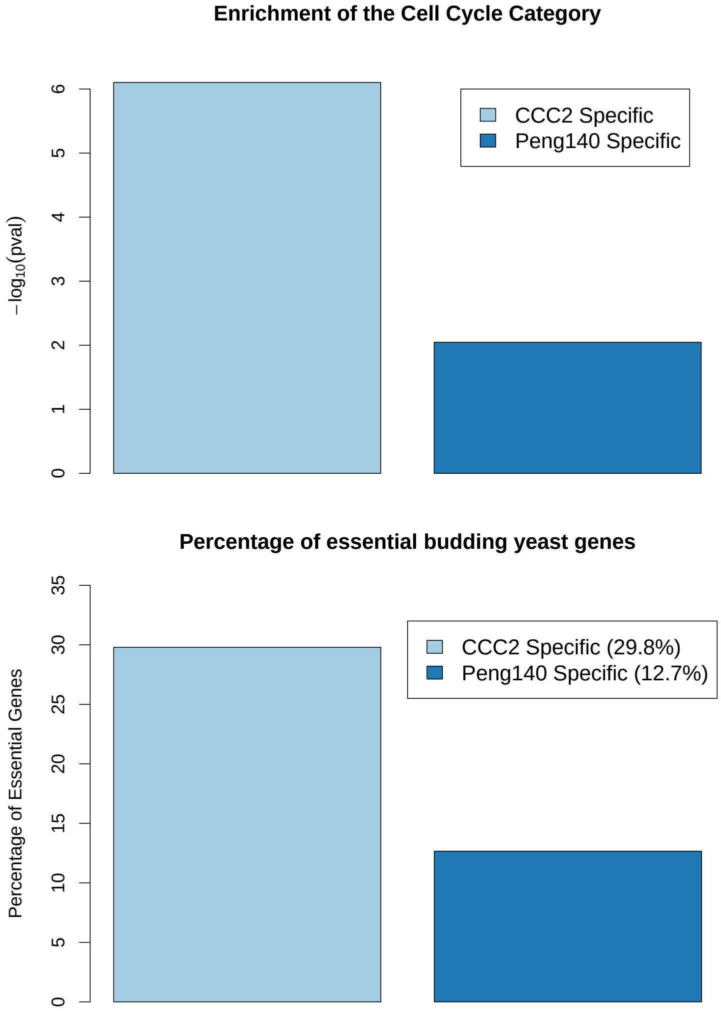

Supporting Figure 19: 136 budding yeast genes were reported to have cycling homologs in fission yeast by Peng *et al.* [6]. Unlike the analysis in our paper which relied on additional species and used a probabilistic algorithm for assigning cycling scores and determining conserved cycling genes, Peng *et al* compared only budding and fission yeast and used a deterministic assignment method . We thus compared the CCC2 list with this list by Peng *et al.* There are 63 genes that are present in both lists, 77 genes unique to CCC2, and 73 genes unique to the list in Peng *et al.* (the latter two are denoted by “Set C” and “Set P” henceforth). **Top:** To compare the genes unique to both lists we first performed GO analysis of these two sets. To account for the influence of homology information in the GO database, we removed from GO annotations inferred from sequence or structural similarity. We find that our set, Set C, has a much higher enrichment of cell cycle genes (pvalue=8*10^-7 for Set C, pvalue=9*10^-3 for Set P). **Bottom:** We have also computed the percentage of essential genes in the two sets (similar to the analysis in Figure 4). Again, Set C has a much higher percentage of essential genes (29.8%) than Set P (12.7%). Combined we believe that this highlights the advantages of the probabilistic method used in our paper.
