# Supplementary material for: Combined analysis reveals a core set of cycling genes
Source: Genome Biol. 2007 Jul 24;8(7):R146. doi: 10.1186/gb-2007-8-7-r146 (PMC2323241; doi:10.1186/gb-2007-8-7-r146)
Supplement: Additional data file 2 — Provided are supporting tables. [file gb-2007-8-7-r146-S2.pdf]

Supporting tables for:

Combined analysis reveals a core set of cycling genes

Yong Lu, Shaun Mahony, Panayiotis V. Benos, Roni Rosenfeld, Itamar Simon, Linda L. Breeden, Ziv Bar-Joseph

Tables **Pages**

Supporting tables 1-3: Overlap between our lists and previous cycling genes lists **2-3**

Supporting tables 4-10: Genes in the CCC4 and CCC3 sets **4-23**

Supporting table 11: Motif scan analysis **24**

Supporting table 12: Enriched budding yeast complexes **25-26**

Supporting tables 13-21: GO analysis of conserved and full lists **27-35**

Supporting Table 1: Overlap between identified cycling genes and previously published lists

Species	Reference	Genes in the list	Number in Overlap	Percent Overlap
Budding Yeast	Spellman *et al.*	790	590	74.7
Budding Yeast	Pramila *et al.*	769	533	69.3
Fission Yeast	Rustici *et al.*	400	268	67
Fission Yeast	Oliva *et al.*	709	315	52.5
Fission Yeast	Peng *et al.*	740	310	51.7
Humans	Whitfield *et al.*	778	376	48.3
Arabidopsis	Menges *et al.*	459	170	37

Supporting Table 1: Overlap between our lists and the published lists. We note that the overlap is not significantly different when from the overlap between the three previously published lists for fission yeast [5]. For each species, we count the number of genes common to our list and the published list(s). Percent of overlap is calculated with respect to the shorter list.

Supporting Table 2: Overlap between CCC4 sets and previously published lists of cycling genes

Species	Reference	Genes in the set	Number in Overlap	Percent Overlap
Budding Yeast	Spellman *et al.*	37	33	89.2
Budding Yeast	Pramila *et al.*	37	35	94.6
Fission Yeast	Rustici *et al.*	39	21	53.8
Fission Yeast	Oliva *et al.*	39	23	59
Fission Yeast	Peng *et al.*	39	23	59
Humans	Whitfield *et al.*	52	33	63.5
Arabidopsis	Menges *et al.*	39	25	64.1

Supporting Table 2: Overlap analysis of the CCC4 set and the published lists. For each species, we count the number of genes common to the CCC4 set and the published list(s). Percent of overlap is calculated with respect to the CCC4 set.

Supporting Table 3: Overlap between CCC3 sets and previously published lists of cycling genes

Species	Reference	Genes in the list	Number in Overlap	Percent Overlap
Budding Yeast	Spellman *et al*.	72	59	81.9
Budding Yeast	Pramila *et al*.	72	64	88.9
Fission Yeast	Rustici *et al*.	68	38	55.9
Fission Yeast	Oliva *et al*.	68	42	61.8
Fission Yeast	Peng *et al*.	68	44	64.7
Humans	Whitfield *et al*.	83	47	56.6

Supporting Table 3: Overlap analysis of the CCC3 set and the published lists. For each species, we count the number of genes common to the CCC3 set and the published list(s). Percent of overlap is calculated with respect to the CCC3 set.

Supporting Table 4: Budding yeast genes in the CCC4 set.

ORF	Symbol	Aliases	Description
YFL039C	ACT1	ABY1|END7	Actin, structural protein involved in cell polarization, endocytosis, and other cytoskeletal functions
YNR044W	AGA1		Anchorage subunit of a-agglutinin of a-cells, highly O-glycosylated protein with N-terminal secretion signal and C-terminal signal for addition of GPI anchor to cell wall, linked to adhesion subunit Aga2p via two disulfide bonds
YPR034W	ARP7	SWP61	Actin-related protein involved in transcriptional regulation; subunit of the chromatin remodeling Snf/Swi complex
YGL116W	CDC20	PAC5	Cell-cycle regulated activator of anaphase-promoting complex/cyclosome (APC/C), which is required for metaphase/anaphase transition; directs ubiquitination of mitotic cyclins, Pds1p, and other anaphase inhibitors; potential Cdc28p substrate
YLR274W	CDC46	BOB1|MCM5	Component of the hexameric MCM complex, which is important for priming origins of DNA replication in G1 and becomes an active ATP-dependent helicase that promotes DNA melting and elongation when activated by Cdc7p-Dbf4p in S-phase
YBR202W	CDC47	MCM7	Component of the hexameric MCM complex, which is important for priming origins of DNA replication in G1 and becomes an active ATP-dependent helicase that promotes DNA melting and elongation when activated by Cdc7p-Dbf4p in S-phase
YPR019W	CDC54	HCD21|MCM4	Essential helicase component of heterohexameric MCM2-7 complexes which bind pre-replication complexes on DNA and melt the DNA prior to replication; accumulates in the nucleus in G1; homolog of S. pombe Cdc21p
YJL194W	CDC6		Essential ATP-binding protein required for DNA replication, component of the pre-replicative complex (pre-RC) which requires ORC to associate with chromatin and is in turn required for Mcm2-7p DNA association; homologous to S. pombe Cdc18p
YEL061C	CIN8	KSL2|SDS15	Kinesin motor protein involved in mitotic spindle assembly and chromosome segregation
YGR108W	CLB1	SCB1	B-type cyclin involved in cell cycle progression; activates Cdc28p to promote the transition from G2 to M phase; accumulates during G2 and M, then targeted via a destruction box motif for ubiquitin-mediated degradation by the proteasome
YPR119W	CLB2		B-type cyclin involved in cell cycle progression; activates Cdc28p to promote the transition from G2 to M phase; accumulates during G2 and M, then targeted via a destruction box motif for ubiquitin-mediated degradation by the proteasome
YLR210W	CLB4		B-type cyclin involved in cell cycle progression; activates Cdc28p to promote the G2/M transition; may be involved in DNA replication and spindle assembly; accumulates during S phase and G2, then targeted for ubiquitin-mediated degradation
YPR120C	CLB5		B-type cyclin involved in DNA replication during S phase; activates Cdc28p to promote initiation of DNA synthesis;

			functions in formation of mitotic spindles along with Clb3p and Clb4p; most abundant during late G1 phase
YGR109C	CLB6		B-type cyclin involved in DNA replication during S phase; activates Cdc28p to promote initiation of DNA synthesis; functions in formation of mitotic spindles along with Clb3p and Clb4p; most abundant during late G1
YKL049C	CSE4	CSL2	Centromere protein that resembles histones, required for proper kinetochore function; homolog of human CENP-A
YCR089W	FIG2		Cell wall adhesin, expressed specifically during mating; may be involved in maintenance of cell wall integrity during mating
YBR010W	HHT1	BUR5|SIN2	One of two identical histone H3 proteins (see also HHT2); core histone required for chromatin assembly, involved in heterochromatin-mediated telomeric and HM silencing; regulated by acetylation, methylation, and mitotic phosphorylation
YNL031C	HHT2		One of two identical histone H3 proteins (see also HHT1); core histone required for chromatin assembly, involved in heterochromatin-mediated telomeric and HM silencing; regulated by acetylation, methylation, and mitotic phosphorylation
YDR225W	HTA1	H2A1|SPT11	One of two nearly identical (see also HTA2) histone H2A subtypes; core histone required for chromatin assembly and chromosome function; DNA damage-dependent phosphorylation by Mec1p facilitates DNA repair; acetylated by Nat4p
YBL003C	HTA2	H2A2	One of two nearly identical (see also HTA1) histone H2A subtypes; core histone required for chromatin assembly and chromosome function; DNA damage-dependent phosphorylation by Mec1p facilitates DNA repair; acetylated by Nat4p
YOL012C	HTZ1	HTA3	Histone variant H2AZ, exchanged for histone H2A in nucleosomes by the SWR1 complex; involved in transcriptional regulation through prevention of the spread of silent heterochromatin
YPR141C	KAR3	OSR11	Minus-end-directed microtubule motor that functions in mitosis and meiosis, localizes to the spindle pole body and localization is dependent on functional Cik1p, required for nuclear fusion during mating; potential Cdc28p substrate
YBL063W	KIP1	CIN9	Kinesin-related motor protein required for mitotic spindle assembly and chromosome segregation; functionally redundant with Cin8p
YBL023C	MCM2		Protein involved in DNA replication; component of the Mcm2-7 hexameric complex that binds chromatin as a part of the pre-replicative complex
YEL032W	MCM3		Protein involved in DNA replication; component of the Mcm2-7 hexameric complex that binds chromatin as a part of the pre-replicative complex
YGL201C	MCM6		Protein involved in DNA replication; component of the Mcm2-7 hexameric complex that binds chromatin as a part of the pre-replicative complex
YIL106W	MOB1		Component of the mitotic exit network; associates with and is required for the activation and Cdc15p-dependent phosphorylation of the Dbf2p kinase; required for cytokinesis and cell separation; component of the CCR4

			transcriptional complex
YHR086W	NAM8	MRE2|MUD15	RNA binding protein, component of the U1 snRNP protein; mutants are defective in meiotic recombination and in formation of viable spores, involved in the formation of DSBs through meiosis-specific splicing of MER2 pre-mRNA
YDR150W	NUM1	PAC12	Protein required for nuclear migration, localizes to the mother cell cortex and the bud tip; may mediate interactions of dynein and cytoplasmic microtubules with the cell cortex
YML065W	ORC1		Largest subunit of the origin recognition complex, which directs DNA replication by binding to replication origins and is also involved in transcriptional silencing; may be phosphorylated by Cdc28p
YOR127W	RGA1	DBM1|THE1	GTPase-activating protein for the polarity-establishment protein Cdc42p; implicated in control of septin organization, pheromone response, and haploid invasive growth
YDR077W	SED1		Major stress-induced structural GPI-cell wall glycoprotein in stationary-phase cells, associates with translating ribosomes, possible role in mitochondrial genome maintenance; ORF contains two distinct variable minisatellites
YJL074C	SMC3		Subunit of the multiprotein cohesin complex required for sister chromatid cohesion in mitotic cells; also required, with Rec8p, for cohesion and recombination during meiosis; phylogenetically conserved SMC chromosomal ATPase family member
YLR045C	STU2		Microtubule-associated protein (MAP) of the XMAP215/Dis1 family; regulates microtubule dynamics during spindle orientation and metaphase chromosome alignment; interacts with spindle pole body component Spc72p
YFL037W	TUB2	ARM10|SHE8	Beta-tubulin; associates with alpha-tubulin (Tub1p and Tub3p) to form tubulin dimer, which polymerizes to form microtubules
YLR212C	TUB4		Gamma-tubulin, involved in nucleating microtubules from both the cytoplasmic and nuclear faces of the spindle pole body
YCR084C	TUP1	AAR1|AER2|AMM1|CRT4|CYC9|FLK1|ROX4|SFL2|UMR7	General repressor of transcription, forms complex with Cyc8p, involved in the establishment of repressive chromatin structure through interactions with histones H3 and H4, appears to enhance expression of some genes

Supporting Table 5: Fission yeast genes in the CCC4 set.

Name	Description
act1	actin
alp14	Mad2-dependent spindle checkpoint component
arp3	actin-like protein
cdc13	cyclin
cdc18	MCM loader
cdc21	MCM complex subunit Cdc21
cig1	cyclin
cig2	cyclin
cnp1	CENP-A
csx1	RNA-binding protein Csx1
cut7	kinesin-like protein Cut7
dis1	microtubule-associated protein Dis1
h3.3	histone H3
hht1	histone H3
hht2	histone H3
hta1	histone H2A
hta2	histone H2A
klp2	kinesin-like protein Klp2
klp3	kinesin-like protein Klp3
klp8	kinesin-like protein Klp8
mcm3	MCM complex subunit Mcm3
mcm5	MCM complex subunit Mcm5
mcm6	MCM complex subunit Mcm6
mob1	protein kinase regulator Mob1
nda2	tubulin alpha 1
nda3	tubulin beta
pht1	histone H2A variant
prp5	WD repeat protein Prp5
psm3	mitotic cohesin complex subunit Psm3
rga2	GTPase activating protein
slp1	sleepy homolog Slp1
SPAC2E1P5.05	U3 snoRNP-associated protein
SPBC15D4.01c	kinesin-like protein
SPBC23E6.01c	RNA-binding protein
SPBC4F6.13c	WD repeat protein
SPBPJ4664.02	glycoprotein
SPCC18.05c	notchless-like protein

SPCC970.10c	ubiquitin-protein ligase E3
srw1	CDK inhibitor Srw1

Supporting Table 6: Human genes in the CCC4 set.

Name	Description
ACTA2	ACTA2, ACTSA, ACTVS: Actin, aortic smooth muscle
ACTR1A	ACTR1A, CTRN1: Alpha-centractin
ACTR2	
ACTR6	ACTR6, CDA12: Actin-related protein 6
ANKRD17	KIAA0697, ANKRD17: KIAA0697 protein (Fragment)
CCNA2	CCNA2, CCN1, CCNA: Cyclin-A2
CCNB1	Cyclin B1
CCNE1	CCNE1, CCNE: G1/S-specific cyclin-E1
CCNF	CCNF: G2/mitotic-specific cyclin-F
CDC20	CDC20: Cell division cycle protein 20 homolog
CDC6	CDC6, CDC18L: Cell division control protein 6 homolog
CENPE	CENPE variant protein: CENPE variant protein (Fragment)
CENPF	CENPF: Centromere protein F
CKAP5	CKAP5: CKAP5 protein (Fragment)
CSPG6	CSPG6, BAM, BMH, SMC3, SMC3L1: Structural maintenance of chromosome 3
ELAVL2	ELAVL2, RP11-315I14.4-004: ELAV
FZR1	FZR1, CDH1, FYR, FZR, KIAA1242: Fizzy-related protein homolog
GRLF1	GRLF1, GRF1, KIAA1722: Glucocorticoid receptor DNA-binding factor 1
H3F3A	NA
HIST1H2AC	HIST1H2AC, H2AFL: Histone H2A type 1-C
HIST2H2AA	HIST2H2AA3, H2AFO, HIST2H2AA: Histone H2A type 2-A
KIF11	KIF11, EG5, KNSL1: Kinesin-like protein KIF11
KIF14	KIF14, KIAA0042: Kinesin-like protein KIF14
KIF1A	KIF1A, ATSV: Kinesin-like protein KIF1A
KIF1B	KIF1B, KIAA0591, KIAA1448: Kinesin-like protein KIF1B
KIF22	KIF22, KID, KNSL4: Kinesin-like protein KIF22
KIF23	KIF23: KIF23 protein (Fragment)
KIF2C	KIF2C, KNSL6: Kinesin-like protein KIF2C
KIF5A	KIF5A, NKHC1: Kinesin heavy chain isoform 5A
KIF5B	
KIF9	KIF9: Kinesin-like protein KIF9
KIFC1	KIFC1, HSET, KNSL2: Kinesin-like protein KIFC1
KIFC3	KIFC3: Kinesin-like protein KIFC3
MCM2	MCM2, BM28, CDCL1, KIAA0030: DNA replication licensing factor MCM2
MCM3	MCM3, RP1-108C2.3-004: MCM3 minichromosome maintenance deficient 3 (Fragment)
MCM4	MCM4, CDC21: DNA replication licensing factor MCM4

MCM5	Minichromosome maintenance deficient protein 5 variant (Fragment)
MCM6	MCM6: DNA replication licensing factor MCM6
MCM8	MCM8, C20orf154: DNA replication licensing factor MCM8
MDC1	MDC1, KIAA0170, NFBD1: Mediator of DNA damage checkpoint protein 1
MOBKL2C	MOBKL2C, RP11-49P4.4-002: MOB1, Mps One Binder kinase activator-like 2C
ORC1L	ORC1L, ORC1, PARC1: Origin recognition complex subunit 1
RNPC3	RNP, RNPC3, RP5-1108M17.6-002: CDNA FLJ25070 fis, clone CBL05164
RNU3IP2	RNU3IP2, U355K: U3 small nucleolar RNA-interacting protein 2
SMC4L1	SMC4L1, CAPC, SMC4: Structural maintenance of chromosomes 4-like 1 protein
TLE3	TLE3, KIAA1547: Transducin-like enhancer protein 3
TUBA1	
TUBA2	TUBA2: Tubulin alpha-2 chain
TUBA3	TUBA3: Tubulin alpha-3 chain
TUBG1	TUBG1, TUBG: Tubulin gamma-1 chain
WDTC1	RP11-4K3__A.1, RP11-4K3__A.1-003: Novel protein
WSB1	WSB1, SWIP1: WD repeat and SOCS box-containing protein 1

Supporting Table 7: *Arabidopsis* genes in the CCC4 set.

Name	Description
ACT1	ACT1 (ACTIN 1); structural constituent of cytoskeleton
ACT11	Actin-11
ACT7	Actin-7
At1g09200	DNA binding
At2g07690	Minichromosome maintenance family protein
At2g17620	Cyclin
At2g21300	Kinesin motor family protein
At2g22610	Kinesin motor protein-related
At2g28620	Kinesin motor protein-related
At2g35410	33 kDa ribonucleoprotein
At2g36200	Probable 125 kDa kinesin-related protein
At2g37420	Kinesin motor protein-related
At2g47500	Putative kinesin
At3g54560	Histone H2A variant 1
At3g54870	Kinesin-like protein
At4g03100	Rac GTPase activator
At4g14330	Kinesin like protein
At4g19050	Mob1/phocein family protein
At4g35110	NA
At4g35620	Cyclin 2b (CYC2b)
At4g39050	Kinesin-related protein (MKRP2), kinesin motor protein - Ustilago maydis, PID:g2062750
At5g26900	WD-40 repeat family protein
ATK3	Kinesin-3
CDC20.2	WD-40 repeat family protein
CDC6	Cell division control protein CDC6
CYC1BAT	Cyclin 1b (CYC1b)
CYCA1;2	Mitotic cyclin a2-type, putative
CYCA2;1	Cyclin 3a
CYCB1;4	Cyclin
CYCB3;1	Cyclin family protein
CYCD1;1	Cyclin delta-1
CYCD4;1	Cyclin
MOR1	Microtubule organization 1 protein (MOR1)
PAKRP1	Phragmoplast-associated kinesin-related protein (PAKRP1)
PEARLI4	PEARLI4
SPA1	Phytochrome A supressor spa1 (SPA1)
TTN7	Similar to SMC2-like condensin

TUB5	Tubulin beta-5 chain
TUB6	Tubulin beta-6 chain

Supporting Table 8: Budding yeast genes in the CCC3 set.

ORF	Symbol	Aliases	Description
YLR131C	ACE2		Transcription factor that activates expression of early G1-specific genes, localizes to daughter cell nuclei after cytokinesis and delays G1 progression in daughters, localization is regulated by phosphorylation; potential Cdc28p substrate
YFL039C	ACT1	ABY1|END7	Actin, structural protein involved in cell polarization, endocytosis, and other cytoskeletal functions
YNR044W	AGA1		Anchorage subunit of a-agglutinin of a-cells, highly O-glycosylated protein with N-terminal secretion signal and C-terminal signal for addition of GPI anchor to cell wall, linked to adhesion subunit Aga2p via two disulfide bonds
YPR034W	ARP7	SWP61	Actin-related protein involved in transcriptional regulation; subunit of the chromatin remodeling Snf/Swi complex
YOR058C	ASE1	YOR29-09	Member of a family of microtubule-associated proteins (MAPs) that function at the mitotic spindle midzone; required for spindle elongation; undergoes cell cycle-regulated degradation by anaphase promoting complex; potential Cdc28p substrate
YGL116W	CDC20	PAC5	Cell-cycle regulated activator of anaphase-promoting complex/cyclosome (APC/C), which is required for metaphase/anaphase transition; directs ubiquitination of mitotic cyclins, Pds1p, and other anaphase inhibitors; potential Cdc28p substrate
YLR274W	CDC46	BOB1|MCM5	Component of the hexameric MCM complex, which is important for priming origins of DNA replication in G1 and becomes an active ATP-dependent helicase that promotes DNA melting and elongation when activated by Cdc7p-Dbf4p in S-phase
YBR202W	CDC47	MCM7	Component of the hexameric MCM complex, which is important for priming origins of DNA replication in G1 and becomes an active ATP-dependent helicase that promotes DNA melting and elongation when activated by Cdc7p-Dbf4p in S-phase
YPR019W	CDC54	HCD21|MCM4	Essential helicase component of heterohexameric MCM2-7 complexes which bind pre-replication complexes on DNA and melt the DNA prior to replication; accumulates in the nucleus in G1; homolog of S. pombe Cdc21p
YJL194W	CDC6		Essential ATP-binding protein required for DNA replication, component of the pre-replicative complex (pre-RC) which requires ORC to associate with chromatin and is in turn required for Mcm2-7p DNA association; homologous to S. pombe Cdc18p
YEL061C	CIN8	KSL2|SDS15	Kinesin motor protein involved in mitotic spindle assembly and chromosome segregation
YGR108W	CLB1	SCB1	B-type cyclin involved in cell cycle progression; activates Cdc28p to promote the transition from G2 to M phase; accumulates during G2 and M, then targeted via a destruction box motif for ubiquitin-mediated degradation by the proteasome
YPR119W	CLB2		B-type cyclin involved in cell cycle progression; activates Cdc28p to promote the transition from G2 to M phase;

			accumulates during G2 and M, then targeted via a destruction box motif for ubiquitin-mediated degradation by the proteasome
YLR210W	CLB4		B-type cyclin involved in cell cycle progression; activates Cdc28p to promote the G2/M transition; may be involved in DNA replication and spindle assembly; accumulates during S phase and G2, then targeted for ubiquitin-mediated degradation
YPR120C	CLB5		B-type cyclin involved in DNA replication during S phase; activates Cdc28p to promote initiation of DNA synthesis; functions in formation of mitotic spindles along with Clb3p and Clb4p; most abundant during late G1 phase
YGR109C	CLB6		B-type cyclin involved in DNA replication during S phase; activates Cdc28p to promote initiation of DNA synthesis; functions in formation of mitotic spindles along with Clb3p and Clb4p; most abundant during late G1
YKL049C	CSE4	CSL2	Centromere protein that resembles histones, required for proper kinetochore function; homolog of human CENP-A
YPR104C	FHL1		Putative transcriptional regulator with similarity to DNA-binding domain of Drosophila forkhead; required for rRNA processing
YCR089W	FIG2		Cell wall adhesin, expressed specifically during mating; may be involved in maintenance of cell wall integrity during mating
YIL131C	FKH1		Transcription factor of the forkhead family that regulates the cell cycle and pseudohyphal growth; also involved in chromatin silencing at HML and HMR
YNL068C	FKH2		Transcription factor of the forkhead family that regulates the cell cycle and pseudohyphal growth; also involved in chromatin silencing at HML and HMR; potential Cdc28p substrate
YCR065W	HCM1		Forkhead transcription factor involved in cell cycle specific transcription of SPC110; dosage-dependent suppressor of calmodulin mutants with specific defects in SPB assembly; involved in telomere maintenance
YBL032W	HEK2	KHD1	RNA binding protein with similarity to hnRNP-K that localizes to the cytoplasm and to subtelomeric DNA; required for the proper localization of ASH1 mRNA; involved in the regulation of telomere position effect and telomere length
YBR009C	HHF1		One of two identical histone H4 proteins (see also HHF2); core histone required for chromatin assembly and chromosome function; contributes to telomeric silencing; N-terminal domain involved in maintaining genomic integrity
YNL030W	HHF2		One of two identical histone H4 proteins (see also HHF1); core histone required for chromatin assembly and chromosome function; contributes to telomeric silencing; N-terminal domain involved in maintaining genomic integrity
YBR010W	HHT1	BUR5|SIN2	One of two identical histone H3 proteins (see also HHT2); core histone required for chromatin assembly, involved in heterochromatin-mediated telomeric and HM silencing; regulated by acetylation, methylation, and mitotic phosphorylation

YNL031C	HHT2		One of two identical histone H3 proteins (see also HHT1); core histone required for chromatin assembly, involved in heterochromatin-mediated telomeric and HM silencing; regulated by acetylation, methylation, and mitotic phosphorylation
YMR032W	HOF1	CYK2	Bud neck-localized, SH3 domain-containing protein required for cytokinesis; regulates actomyosin ring dynamics and septin localization; interacts with the formins, Bni1p and Bnr1p, and with Cyk3p, Vrp1p, and Bni5p
YDR225W	HTA1	H2A1|SPT11	One of two nearly identical (see also HTA2) histone H2A subtypes; core histone required for chromatin assembly and chromosome function; DNA damage-dependent phosphorylation by Mec1p facilitates DNA repair; acetylated by Nat4p
YBL003C	HTA2	H2A2	One of two nearly identical (see also HTA1) histone H2A subtypes; core histone required for chromatin assembly and chromosome function; DNA damage-dependent phosphorylation by Mec1p facilitates DNA repair; acetylated by Nat4p
YDR224C	HTB1	SPT12	One of two nearly identical (see HTB2) histone H2B subtypes required for chromatin assembly and chromosome function; Rad6p-Bre1p-Lge1p mediated ubiquitination regulates transcriptional activation, meiotic DSB formation and H3 methylation
YBL002W	HTB2		One of two nearly identical (see HTB1) histone H2B subtypes required for chromatin assembly and chromosome function; Rad6p-Bre1p-Lge1p mediated ubiquitination regulates transcriptional activation, meiotic DSB formation and H3 methylation
YOL012C	HTZ1	HTA3	Histone variant H2AZ, exchanged for histone H2A in nucleosomes by the SWR1 complex; involved in transcriptional regulation through prevention of the spread of silent heterochromatin
YPL242C	IQG1	CYK1	Essential protein required for determination of budding pattern, promotes localization of axial markers Bud4p and Cdc12p and functionally interacts with Sec3p, localizes to the contractile ring during anaphase, member of the IQGAP family
YPR141C	KAR3	OSR11	Minus-end-directed microtubule motor that functions in mitosis and meiosis, localizes to the spindle pole body and localization is dependent on functional Cik1p, required for nuclear fusion during mating; potential Cdc28p substrate
YGR238C	KEL2		Protein that functions in a complex with Kel1p to negatively regulate mitotic exit, interacts with Tem1p and Lte1p; localizes to regions of polarized growth; potential Cdc28p substrate
YBL063W	KIP1	CIN9	Kinesin-related motor protein required for mitotic spindle assembly and chromosome segregation; functionally redundant with Cin8p
YKL008C	LAC1	DGT1	Ceramide synthase component, involved in synthesis of ceramide from C26(acyl)-coenzyme A and dihydrosphingosine or phytosphingosine, functionally equivalent to Lag1p
YBL023C	MCM2		Protein involved in DNA replication; component of the

			Mcm2-7 hexameric complex that binds chromatin as a part of the pre-replicative complex
YEL032W	MCM3		Protein involved in DNA replication; component of the Mcm2-7 hexameric complex that binds chromatin as a part of the pre-replicative complex
YGL201C	MCM6		Protein involved in DNA replication; component of the Mcm2-7 hexameric complex that binds chromatin as a part of the pre-replicative complex
YIL106W	MOB1		Component of the mitotic exit network; associates with and is required for the activation and Cdc15p-dependent phosphorylation of the Dbf2p kinase; required for cytokinesis and cell separation; component of the CCR4 transcriptional complex
YOL112W	MSB4		GTPase-activating protein of the Ras superfamily that acts primarily on Sec4p, localizes to the bud site and bud tip, has similarity to Msb3p; msb3 msb4 double mutation causes defects in secretion and actin organization
YOL090W	MSH2	PMS5	Protein that forms heterodimers with Msh3p and Msh6p that bind to DNA mismatches to initiate the mismatch repair process; contains a Walker ATP-binding motif required for repair activity; Msh2p-Msh6p binds to and hydrolyzes ATP
YDR097C	MSH6	PMS3	Protein required for mismatch repair in mitosis and meiosis, forms a complex with Msh2p to repair both single-base & insertion-deletion mispairs; potentially phosphorylated by Cdc28p
YPL247C	NA	NA	NA
YHR086W	NAM8	MRE2|MUD15	RNA binding protein, component of the U1 snRNP protein; mutants are defective in meiotic recombination and in formation of viable spores, involved in the formation of DSBs through meiosis-specific splicing of MER2 pre-mRNA
YDR150W	NUM1	PAC12	Protein required for nuclear migration, localizes to the mother cell cortex and the bud tip; may mediate interactions of dynein and cytoplasmic microtubules with the cell cortex
YML065W	ORC1		Largest subunit of the origin recognition complex, which directs DNA replication by binding to replication origins and is also involved in transcriptional silencing; may be phosphorylated by Cdc28p
YBR233W	PBP2	HEK1	RNA binding protein with similarity to mammalian heterogeneous nuclear RNP K protein, involved in the regulation of telomere position effect and telomere length
YMR076C	PDS5		Protein required for establishment and maintenance of sister chromatid condensation and cohesion, colocalizes with cohesin on chromosomes in an interdependent manner, may function as a protein-protein interaction scaffold
YDR481C	PHO8		Repressible alkaline phosphatase, a glycoprotein localized to the vacuole; regulated by levels of inorganic phosphate and by a system consisting of Pho4p, Pho9p, Pho80p, Pho81p and Pho85p; dephosphorylates phosphotyrosyl peptides
YNL262W	POL2	DUN2	Catalytic subunit of DNA polymerase epsilon, one of the major chromosomal DNA replication polymerases

			characterized by processivity and proofreading exonuclease activity; also involved in DNA synthesis during DNA repair
YKL045W	PRI2		Subunit of DNA primase, which is required for DNA synthesis and double-strand break repair
YER095W	RAD51	MUT5	Strand exchange protein, forms a helical filament with DNA that searches for homology; involved in the recombinational repair of double-strand breaks in DNA during vegetative growth and meiosis; homolog of Dmc1p and bacterial RecA protein
YOR127W	RGA1	DBM1|THE1	GTPase-activating protein for the polarity-establishment protein Cdc42p; implicated in control of septin organization, pheromone response, and haploid invasive growth
YPR165W	RHO1		GTP-binding protein of the rho subfamily of Ras-like proteins, involved in establishment of cell polarity; regulates protein kinase C (Pkc1p) and the cell wall synthesizing enzyme 1,3-beta-glucan synthase (Fks1p and Gsc2p)
YER070W	RNR1	CRT7|RIR1| SDS12	Ribonucleotide-diphosphate reductase (RNR), large subunit; the RNR complex catalyzes the rate-limiting step in dNTP synthesis and is regulated by DNA replication and DNA damage checkpoint pathways via localization of the small subunits
YIL066C	RNR3	DIN1|RIR3	Ribonucleotide-diphosphate reductase (RNR), large subunit; the RNR complex catalyzes the rate-limiting step in dNTP synthesis and is regulated by DNA replication and DNA damage checkpoint pathways via localization of the small subunits
YGR152C	RSR1	BUD1	GTP-binding protein of the ras superfamily required for bud site selection, morphological changes in response to mating pheromone, and efficient cell fusion; localized to the plasma membrane; significantly similar to mammalian Rap GTPases
YDR077W	SED1		Major stress-induced structural GPI-cell wall glycoprotein in stationary-phase cells, associates with translating ribosomes, possible role in mitochondrial genome maintenance; ORF contains two distinct variable minisatellites
YJL074C	SMC3		Subunit of the multiprotein cohesin complex required for sister chromatid cohesion in mitotic cells; also required, with Rec8p, for cohesion and recombination during meiosis; phylogenetically conserved SMC chromosomal ATPase family member
YHR050W	SMF2		Divalent metal ion transporter involved in manganese homeostasis; has broad specificity for di-valent and tri-valent metals; post-translationally regulated by levels of metal ions; member of the Nramp family of metal transport proteins
YLR034C	SMF3		Putative divalent metal ion transporter involved in iron homeostasis; transcriptionally regulated by metal ions; member of the Nramp family of metal transport proteins
YLR045C	STU2		Microtubule-associated protein (MAP) of the XMAP215/Dis1 family; regulates microtubule dynamics during spindle orientation and metaphase chromosome

			alignment; interacts with spindle pole body component Spc72p
YJL187C	SWE1	WEE1	Protein kinase that regulates the G2/M transition by inhibition of Cdc28p kinase activity; localizes to the nucleus and to the daughter side of the mother-bud neck; homolog of S. pombe Wee1p; potential Cdc28p substrate
YDR146C	SWI5		Transcription factor that activates transcription of genes expressed in G1 phase and at the G1/M boundary; localization to the nucleus occurs during G1 and appears to be regulated by phosphorylation by Cdc28p kinase
YLR136C	TIS11	CTH2	mRNA-binding protein expressed during iron starvation; binds to a sequence element in the 3'-untranslated regions of specific mRNAs to mediate their degradation; involved in iron homeostasis
YFL037W	TUB2	ARM10|SHE8	Beta-tubulin; associates with alpha-tubulin (Tub1p and Tub3p) to form tubulin dimer, which polymerizes to form microtubules
YLR212C	TUB4		Gamma-tubulin, involved in nucleating microtubules from both the cytoplasmic and nuclear faces of the spindle pole body
YCR084C	TUP1	AAR1|AER2| AMM1|CRT4| CYC9|FLK1| ROX4|SFL2| UMR7	General repressor of transcription, forms complex with Cyc8p, involved in the establishment of repressive chromatin structure through interactions with histones H3 and H4, appears to enhance expression of some genes
YBL004W	UTP20		Component of the small-subunit (SSU) processome, which is involved in the biogenesis of the 18S rRNA

Supporting Table 9: Fission yeast genes in the CCC3 set.

**Name**	**Description**
ace2	transcription factor Ace2
act1	actin
alp14	Mad2-dependent spindle checkpoint component
arp3	actin-like protein
ase1	microtubule-associated protein Ase1
cdc13	cyclin
cdc15	cell division control protein Cdc15
cdc18	MCM loader
cdc20	DNA polymerase epsilon catalytic subunit a Pol2
cdc21	MCM complex subunit Cdc21
cdc22	ribonucleoside reductase large subunit Cdc22
cdc42	Rho family GTPase Cdc42
cig1	cyclin
cig2	cyclin
cnp1	CENP-A
csx1	RNA-binding protein Csx1
cut7	kinesin-like protein Cut7
dis1	microtubule-associated protein Dis1
fkh2	fork head transcription factor Fkh2
h3.3	histone H3
h4.3	histone H4
hhf1	histone H4
hhf2	histone H4
hht1	histone H3
hht2	histone H3
hta1	histone H2A
hta2	histone H2A
htb1	histone H2B
imp2	FCH domain
klp2	kinesin-like protein Klp2
klp3	kinesin-like protein Klp3
klp8	kinesin-like protein Klp8
mcm3	MCM complex subunit Mcm3
mcm5	MCM complex subunit Mcm5
mcm6	MCM complex subunit Mcm6
mik1	mitotic inhibitor kinase Mik1
mob1	protein kinase regulator Mob1

msh6	MutS protein homolog
nda2	tubulin alpha 1
nda3	tubulin beta
pds5	cohesin-associated protein
pht1	histone H2A variant
prp5	WD repeat protein Prp5
psm3	mitotic cohesin complex subunit Psm3
rga2	GTPase activating protein
rho4	Rho family GTPase Rho4
rhp51	recombinase Rhp51
rnc1	RNA-binding protein that suppresses calcineurin deletion Rnc1
rng2	IQGAP
slp1	sleepy homolog Slp1
SPAC27F1.08	Nramp family
SPAC2E1P5.05	U3 snoRNP-associated protein
SPBC14F5.13c	alkaline phosphatase
SPBC15D4.01c	kinesin-like protein
SPBC17D11.08	WD repeat protein
SPBC215.01	GTPase activating protein
SPBC23E6.01c	RNA-binding protein
SPBC3E7.15c	sphingosine N-acyltransferase
SPBC4F6.13c	WD repeat protein
SPBC56F2.04	snoRNA binding
SPBPJ4664.02	glycoprotein
SPCC18.05c	notchless-like protein
SPCC970.10c	ubiquitin-protein ligase E3
spp2	DNA primase large subunit Spp2
srw1	CDK inhibitor Srw1
tea1	cell end marker Tea1
tea3	cell end marker Tea3
zfs1	transcription factor Zfs1

Supporting Table10: Human genes in the CCC3 set.

Name	Description
ACTA2	ACTA2, ACTSA, ACTVS: Actin, aortic smooth muscle
ACTR1A	ACTR1A, CTRN1: Alpha-centractin
ACTR2	
ACTR6	ACTR6, CDA12: Actin-related protein 6
ALPP	ALPP, PLAP: Alkaline phosphatase, placental type precursor
ANKRD17	KIAA0697, ANKRD17: KIAA0697 protein (Fragment)
CCNA2	CCNA2, CCN1, CCNA: Cyclin-A2
CCNB1	Cyclin B1
CCNE1	CCNE1, CCNE: G1/S-specific cyclin-E1
CCNF	CCNF: G2/mitotic-specific cyclin-F
CDC20	CDC20: Cell division cycle protein 20 homolog
CDC42	BB1: Growth-regulating protein BB1
CDC6	CDC6, CDC18L: Cell division control protein 6 homolog
CENPE	CENPE variant protein: CENPE variant protein (Fragment)
CENPF	CENPF: Centromere protein F
CKAP5	CKAP5: CKAP5 protein (Fragment)
CSPG6	CSPG6, BAM, BMH, SMC3, SMC3L1: Structural maintenance of chromosome 3
DRIM	UTP20, DRIM: Small subunit processome component 20 homolog
ELAVL2	ELAVL2, RP11-315I14.4-004: ELAV
FOXK2	
FOXM1	
FZR1	FZR1, CDH1, FYR, FZR, KIAA1242: Fizzy-related protein homolog
GRLF1	GRLF1, GRF1, KIAA1722: Glucocorticoid receptor DNA-binding factor 1
H3F3A	NA
HIST1H2AC	HIST1H2AC, H2AFL: Histone H2A type 1-C
HIST1H2BD	HIST2H2BF: HIST2H2BF protein
HIST1H4I	NA
HIST2H2AA	HIST2H2AA3, H2AFO, HIST2H2AA: Histone H2A type 2-A
HIST2H2BE	
IQGAP2	IQGAP2: Ras GTPase-activating-like protein IQGAP2
IQGAP3	IQGAP3: Ras GTPase-activating-like protein IQGAP3
KIF11	KIF11, EG5, KNSL1: Kinesin-like protein KIF11
KIF14	KIF14, KIAA0042: Kinesin-like protein KIF14
KIF1A	KIF1A, ATSV: Kinesin-like protein KIF1A
KIF1B	KIF1B, KIAA0591, KIAA1448: Kinesin-like protein KIF1B
KIF22	KIF22, KID, KNSL4: Kinesin-like protein KIF22
KIF23	KIF23: KIF23 protein (Fragment)

KIF2C	KIF2C, KNSL6: Kinesin-like protein KIF2C
KIF5A	KIF5A, NKHC1: Kinesin heavy chain isoform 5A
KIF5B	
KIF9	KIF9: Kinesin-like protein KIF9
KIFC1	KIFC1, HSET, KNSL2: Kinesin-like protein KIFC1
KIFC3	KIFC3: Kinesin-like protein KIFC3
KLF13	KLF13, BTEB3, NSLP1: Krueppel-like factor 13
LASS6	LASS6: LASS6 protein
MCM2	MCM2, BM28, CDCL1, KIAA0030: DNA replication licensing factor MCM2
MCM3	MCM3, RP1-108C2.3-004: MCM3 minichromosome maintenance deficient 3 (Fragment)
MCM4	MCM4, CDC21: DNA replication licensing factor MCM4
MCM5	Minichromosome maintenance deficient protein 5 variant (Fragment)
MCM6	MCM6: DNA replication licensing factor MCM6
MCM8	MCM8, C20orf154: DNA replication licensing factor MCM8
MDC1	MDC1, KIAA0170, NFBD1: Mediator of DNA damage checkpoint protein 1
MOBKL2C	MOBKL2C, RP11-49P4.4-002: MOB1, Mps One Binder kinase activator-like 2C
MSH2	MSH2: DNA mismatch repair protein Msh2
MSH5	MSH5, XXbac-BCX40G17.5-008, XXbac-BPG32J3.7-008: MutS homolog 5 (Fragment)
ORC1L	ORC1L, ORC1, PARC1: Origin recognition complex subunit 1
PCBP2	PCBP2: PCBP2 protein
PKMYT1	PKMYT1, MYT1: Membrane-associated tyrosine- and threonine-specific cdc2-inhibitory kinase
POLE	DNA polymerase epsilon catalytic subunit variant (Fragment)
PRC1	PRC1: Protein regulator of cytokinesis 1
PRIM2A	PREDICTED: similar to DNA primase large subunit, 58kDa
PSTPIP1	PSTPIP1, CD2BP1: Proline-serine-threonine phosphatase-interacting protein 1
RABEPK	p40: P40
RAD51	CDNA FLJ16262 fis, clone IMR322008651, highly similar to DNA REPAIR PROTEIN RAD51
RHOB	RHOB, ARH6, ARHB: Rho-related GTP-binding protein RhoB precursor
RHOH	RHOH, ARHH, TTF: Rho-related GTP-binding protein RhoH
RNPC3	RNP, RNPC3, RP5-1108M17.6-002: CDNA FLJ25070 fis, clone CBL05164
RNU3IP2	RNU3IP2, U355K: U3 small nucleolar RNA-interacting protein 2
RRM1	RRM1, RR1: Ribonucleoside-diphosphate reductase large subunit
SCC-112	KIAA0648: KIAA0648 protein (Fragment)
SLC11A2	SLC11A2: Divalent metal transporter (Fragment)
SMC4L1	SMC4L1, CAPC, SMC4: Structural maintenance of chromosomes 4-like 1 protein
TLE3	TLE3, KIAA1547: Transducin-like enhancer protein 3
TUBA1	
TUBA2	TUBA2: Tubulin alpha-2 chain

TUBA3	TUBA3: Tubulin alpha-3 chain
TUBG1	TUBG1, TUBG: Tubulin gamma-1 chain
USP6NL	USP6NL, KIAA0019: USP6 N-terminal-like protein
WDR68	WDR68, HAN11: WD repeat protein 68
WDTC1	RP11-4K3__A.1, RP11-4K3__A.1-003: Novel protein
WSB1	WSB1, SWIP1: WD repeat and SOCS box-containing protein 1
XRCC3	XRCC3: DNA-repair protein XRCC3
ZFP36L1	ZFP36L1, BERG36, BRF1, ERF1, TIS11B: Butyrate response factor 1

Supporting Table 11: Percentage of input promoter datasets matching appropriate Harbison motif

Dataset	*Fission Cell Cycle*	*Fission Non-Cell Cycle*	*Budding Cell Cycle Conserved*	*Budding Cell Cycle All*
SWI4	29.73%	26.06%	60.71%	55.56%
SWI6	51.43%	30.46%	60%	50.54%
MBP1	52.27%	16.57%	51.72%	45.98%
FKH1	21.95%	25%	52.38%	36.23%
FKH2	32.5%	13.43%	39.13%	40%
NDD1	17.65%	31.48%	50%	44.9%
MCM1	26.92%	15.38%	73.33%	69.05%
ACE2	28.57%	29.63%	33.33%	48.48%
SWI5	50%	44.44%	25%	38%
YOX1	25%	12.5%	42.86%	57.14%
YHP1	0%	0%	50%	33.33%

Supporting Table 11: All promoters were scanned using the appropriate Harbison motifs. In order to set a threshold, 10Mbp of random sequence was generated using the same GC-content as budding or fission yeast. Next we set a threshold for each motif as that which gives a false discovery rate (FDR) of 0.0001 in these random datasets. As can be seen from the table, for a number of factors in G1/S and for Fkh2 the conserved cycling fission yeast genes contained substantially more motifs than the negative control set. The percentage of these motifs for the cycling fission yeast genes was similar to the percentage in the budding yeast sets indicating that their activity is highly conserved between the two species. See supporting Methods for more details.

Supporting Table 12. Enriched budding yeast complexes (Krogan *et al.* [18])

complex id	pval	genes in CCC4	genes in CCC3	complex subunits
54	0.000019	3	0	ATE1, SIC1, CKS1, ***CLB5,*** YLR187W, ***CLB4,*** CLB3, CDC28, ***CLB2***
15	0.006971	2	4	NUP188, PSY2, ***HTB2,*** ARG82, HEX3, TOP2, YDL156W, YBL046W, STP1, ***HHF2, HTB1,*** SPT5, CKI1, ***HHF1,*** ABF2, RRD1, ***HTA2,*** SLX8, PPH3, ***HTA1***
3	0.018395	2	0	SCL1, PRE6, PRE2, YJL132W, ***MCM6,*** PRE5, YKR070W, UMP1, DCG1, SNQ2, TOS8, YDL203C, PRE7, MLP1, PRE10, PMD1, PMT4, SGA1, PRE4, PRE1, GNA1, PUP1, YLR290C, PRE3, PRE8, ***CIN8,*** PUP2, YLR211C, YDR179W-A, PUP3, PRE9, RFT1, YCR076C
251	0.01898	1	0	SKO1, ***HHT1,*** VAC7
285	0.01898	1	0	SPC97, ***TUB4,*** SPC72
303	0.01898	1	0	***STU2,*** TUB3, TUB1
346	0.01898	1	0	***CDC6,*** HSF1, YLR419W
349	0.01898	1	0	PRP22, ***KIP1,*** SOK2
176	0.025229	1	0	***SMC3,*** SMC1, IRR1, MCD1
182	0.025229	1	0	GIC1, YIL067C, ***FIG2,*** MRP13
86	0.043743	1	0	YPL183C, YSC85, CUE5, YMR259C, TRM7, YSC84, ***CLB1***
64	0.049838	1	1	***POL2, MCM2,*** INM1, DPB4, DPB3, MBA1, DPB2, RPN4
412	0.000035	0	2	***MSH6, MSH2***
199	0.00021	0	2	RNR4, ***RNR1,*** RNR2, ***RNR3***
138	0.000348	0	2	SWP1, ***ACE2,*** YIL108W, YNL254C, ***FKH2***
50	0.006387	1	1	***ORC1,*** ORC2, ***SWE1,*** HAT2, YJR154W, ORC6, HAT1, ORC5, ORC3, ORC4
546	0.012009	0	1	***RAD51,*** RAD52
255	0.017961	0	1	YLR089C, ***YPL247C,*** HSP60

231	0.023877	0	1	YER184C, KIP2, ***KEL2,*** KEL1
131	0.02976	0	1	RCL1, BPL1, BMS1, RNT1, ***SMF3***
144	0.02976	0	1	***ASE1,*** STM1, YPL150W, RPS25B, VTS1
98	0.035608	0	1	VPS25, VPS36, ***HCM1,*** YBR025C, SNF8, ADH7
69	0.047201	0	1	YJL192C, MSS1, YTM1, YGR067C, ***PDS5,*** YML059C, NOP7, STP3

Supporting Table 13: GO enrichment comparison for budding yeast; Categories that are significant in both conserved and full sets

Intersection of top 20 enriched GO terms	pval for CCC3	pval for CCC3 (corrected)	pval for general set	pval for general set (corrected)
mitotic cell cycle	5.60E-18	0.001	7.20E-17	0.001
cell cycle	3.10E-15	0.001	1.10E-12	0.001
DNA metabolism	5.60E-15	0.001	1.80E-09	0.001
DNA replication	2.00E-13	0.001	1.20E-13	0.001
DNA-dependent DNA replication	2.70E-12	0.001	7.20E-10	0.001
microtubule-based process	7.40E-10	0.001	1.40E-15	0.001
microtubule cytoskeleton organization and biogenesis	1.70E-09	0.001	2.00E-12	0.001
regulation of cyclin-dependent protein kinase activity	2.50E-09	0.001	1.20E-07	0.001
DNA unwinding during replication	2.50E-09	0.001	1.20E-07	0.001

Supporting Table 13. GO enrichment comparison between conserved set and the whole set of cycling genes in budding yeast

Supporting Table 14: GO enrichment comparison for budding yeast; Categories that are more significant in conserved set

Top 20 enriched GO terms for CCC3 but not for the general set	pval for CCC3	pval for CCC3 (corrected)	pval for general set	pval for general set (corrected)
chromatin assembly or disassembly	2.60E-12	0.001	6.50E-06	0.001
organelle organization and biogenesis	7.30E-12	0.001	0.33	1
pre-replicative complex formation and maintenance	5.40E-11	0.001	8.40E-05	0.022
DNA packaging	6.70E-10	0.001	0.03	0.998
establishment and/or maintenance of chromatin architecture	6.70E-10	0.001	0.03	0.998
cellular physiological process	1.30E-09	0.001	0.23	1
DNA replication initiation	1.80E-09	0.001	1.30E-05	0.002
DNA geometric change	2.50E-09	0.001	1.20E-07	0.001
cytoskeleton organization and biogenesis	2.90E-09	0.001	2.50E-07	0.001
interphase	4.40E-09	0.001	1.10E-05	0.001
interphase of mitotic cell cycle	4.40E-09	0.001	1.10E-05	0.001

Supporting Table 14. GO enrichment comparison between conserved set and the whole set of cycling genes in budding yeast

Supporting Table 15: GO enrichment comparison for budding yeast; Categories that are more significant in full set

Top 20 enriched GO terms for the general set but not for CCC3	pval for CCC3	pval for CCC3 (corrected)	pval for general set	pval for general set (corrected)
mitotic sister chromatid cohesion			4.70E-09	0.001
mitotic sister chromatid segregation	3.70E-06	0.001	6.60E-09	0.001
sister chromatid segregation	4.20E-06	0.001	1.10E-08	0.001
mitotic spindle organization and biogenesis in nucleus	3.40E-06	0.001	1.40E-08	0.001
M phase	1.80E-08	0.001	6.10E-08	0.001
conjugation with cellular fusion	0.0019	0.124	8.20E-08	0.001
sexual reproduction	0.0019	0.124	8.20E-08	0.001
conjugation	0.0019	0.124	8.20E-08	0.001

Supporting Table 15. GO enrichment comparison between conserved set and the whole set of cycling genes in budding yeast

Supporting Table 16: GO enrichment comparison for fission yeast; Categories that are significant in both conserved and full sets

Intersection of top 20 enriched GO terms	pval for CCC3	pval for CCC3 (corrected)	pval for general set	pval for general set (corrected)
organelle organization and biogenesis	6.40E-16	0.001	1.70E-06	0.001
mitotic cell cycle	1.60E-13	0.001	2.60E-11	0.001
cell organization and biogenesis	3.50E-10	0.001	2.90E-07	0.001
cell division	8.00E-10	0.001	4.80E-07	0.001
cytokinesis	8.00E-10	0.001	4.80E-07	0.001
interphase of mitotic cell cycle	2.30E-09	0.001	5.30E-08	0.001
interphase	2.90E-09	0.001	8.70E-08	0.001

Supporting Table 16. GO enrichment comparison between conserved set and the whole set of cycling genes in fission yeast

Supporting Table 17: GO enrichment comparison for fission yeast; Categories that are more significant in conserved set

Top 20 enriched GO terms for CCC3 but not for the general set	pval for CCC3	pval for CCC3 (corrected)	pval for general set	pval for general set (corrected)
cytoskeleton organization and biogenesis	3.10E-11	0.001	0.0059	0.728
nucleosome assembly	3.40E-11	0.001	0.00066	0.17
DNA metabolism	9.90E-10	0.001	0.02	0.982
microtubule-based process	1.00E-09	0.001	0.01	0.916
chromatin assembly or disassembly	1.20E-09	0.001	0.01	0.928
cell cycle	1.30E-09	0.001	9.30E-05	0.012
microtubule-based movement	1.50E-09	0.001	8.00E-04	0.186
cytoskeleton-dependent intracellular transport	2.10E-09	0.001	0.0011	0.276
regulation of cellular process	7.90E-09	0.001	0.0016	0.364
regulation of cellular physiological process	1.90E-08	0.001	0.0012	0.29
chromosome organization and biogenesis	2.10E-08	0.001	0.01	0.902
regulation of physiological process	2.20E-08	0.001	0.0011	0.274
protein complex assembly	2.60E-08	0.001	0.13	1

Supporting Table 17. GO enrichment comparison between conserved set and the whole set of cycling genes in fission yeast

Supporting Table 18: GO enrichment comparison for fission yeast; Categories that are more significant in full set

Top 20 enriched GO terms for the general set but not for CCC3	pval for CCC3	pval for CCC3 (corrected)	pval for general set	pval for general set (corrected)
M phase of mitotic cell cycle	1.50E-05	0.002	6.10E-08	0.001
mitosis	0.00016	0.012	3.60E-07	0.001
cytoplasm organization and biogenesis			3.00E-06	0.001
ribosome biogenesis and assembly			3.00E-06	0.001
ribosome biogenesis			3.50E-06	0.001
cell wall organization and biogenesis			3.50E-06	0.001
external encapsulating structure organization and biogenesis			3.50E-06	0.001
cellular polysaccharide metabolism			5.60E-06	0.001
polysaccharide metabolism			7.60E-06	0.001
DNA replication initiation	1.80E-06	0.001	8.30E-06	0.001
cell wall biosynthesis (sensu Fungi)			1.20E-05	0.002
biopolymer biosynthesis			1.50E-05	0.002
polysaccharide biosynthesis			1.50E-05	0.002

Supporting Table 18. GO enrichment comparison between conserved set and the whole set of cycling genes in fission yeast

Supporting Table 19: GO enrichment comparison for human genes; Categories that are significant in both conserved and full sets

Intersection of top 20 enriched GO terms	pval for CCC3	pval for CCC3 (corrected)	pval for general set	pval for general set (corrected)
microtubule-based movement	1.80E-25	0.001	2.70E-07	0.001
cytoskeleton-dependent intracellular transport	2.10E-23	0.001	1.20E-05	0.01
microtubule-based process	4.30E-22	0.001	7.10E-09	0.001
cell cycle	4.60E-17	0.001	9.30E-12	0.001
DNA-dependent DNA replication	1.60E-15	0.001	2.70E-08	0.001
organelle organization and biogenesis	7.20E-15	0.001	6.50E-05	0.028
mitotic cell cycle	8.00E-15	0.001	1.40E-11	0.001
DNA replication	6.60E-14	0.001	7.10E-08	0.001
M phase	1.80E-13	0.001	4.10E-10	0.001
mitosis	4.90E-12	0.001	3.80E-10	0.001
M phase of mitotic cell cycle	5.70E-12	0.001	5.30E-10	0.001
DNA metabolism	5.70E-12	0.001	1.10E-06	0.002
cell division	1.80E-09	0.001	7.50E-07	0.001
microtubule cytoskeleton organization and biogenesis	1.90E-06	0.001	7.70E-05	0.03

Supporting Table 19. GO enrichment comparison between conserved set and the whole set of cycling genes in humans

Supporting Table 20: GO enrichment comparison for human genes; Categories that are more significant in conserved set

Top 20 enriched GO terms for CCC3 but not for the general set	pval for CCC3	pval for CCC3 (corrected)	pval for general set	pval for general set (corrected)
cytoskeleton organization and biogenesis	1.70E-14	0.001	0.00028	0.108
DNA replication initiation	5.90E-13	0.001	0.00018	0.074
establishment of cellular localization	3.80E-10	0.001	0.03	0.992
cellular localization	4.90E-10	0.001	0.03	0.994
cell organization and biogenesis	1.80E-09	0.001	0.0019	0.404
intracellular transport	2.20E-09	0.001	0.08	1

Supporting Table 20. GO enrichment comparison between conserved set and the whole set of cycling genes in humans

Supporting Table 21: GO enrichment comparison for human genes; Categories that are more significant in the full set

Top 20 enriched GO terms for the general set but not for CCC3	pval for CCC3	pval for CCC3 (corrected)	pval for general set	pval for general set (corrected)
spindle organization and biogenesis			2.30E-08	0.001
chromosome segregation			9.00E-08	0.001
regulation of progression through cell cycle	3.10E-06	0.001	1.40E-07	0.001
regulation of cell cycle	3.20E-06	0.001	1.70E-07	0.001
mitotic spindle organization and biogenesis			9.30E-07	0.002
DNA repair	0.00059	0.058	7.30E-05	0.028

Supporting Table 21. GO enrichment comparison between conserved set and the whole set of cycling genes in human
