# Supplementary material for: Combined analysis reveals a core set of cycling genes
Source: Genome Biol. 2007 Jul 24;8(7):R146. doi: 10.1186/gb-2007-8-7-r146 (PMC2323241; doi:10.1186/gb-2007-8-7-r146)
Supplement: Additional data file 3 — Provided are supporting methodologic details. [file gb-2007-8-7-r146-S3.pdf]

Supporting Methods and Results for:

Combined analysis reveals a core set of cycling genes

Yong Lu, Shaun Mahony, Panayiotis V. Benos, Roni Rosenfeld, Itamar Simon, Linda L. Breeden, Ziv Bar-Joseph

1. Probabilistic model for identifying cell cycle genes

We built our probabilistic model for identifying cell cycle genes by following the method in [12]. The model provides a framework to integrate both sequence data and microarray data. We used Markov random fields to represent dependencies between genes in different species. There are two types of nodes in the graph (Figure 1). The first represents genes and the second represents expression scores from the related cell cycle experiments. Edges between gene nodes correspond to sequence similarity, and carry a weight which depends on that similarity. These edges are used to capture the conditional dependencies of phylogenetically related genes. The details on how we generated edges between potential homologous genes are described in section 2.

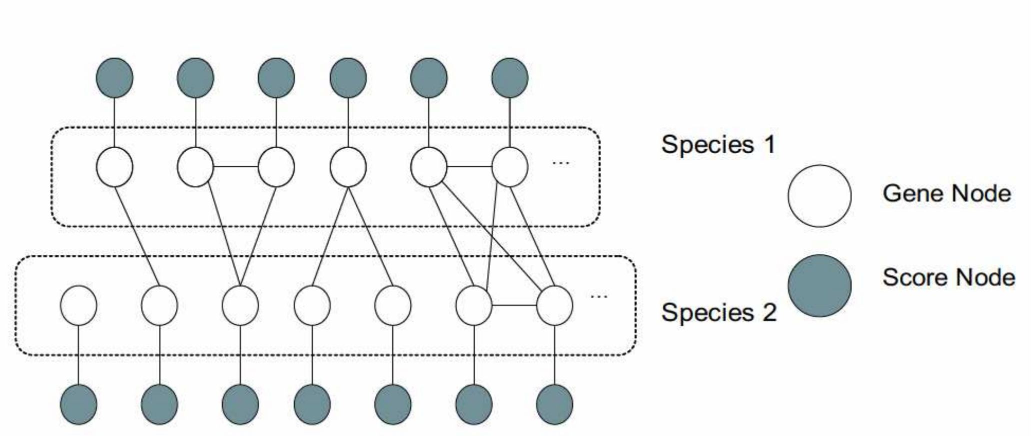

Figure. 1. A graphical model for two species. Dark nodes are score nodes, representing the score derived from experiments. The lighter nodes are gene nodes. Gene nodes are connected by edges if their sequence is similar.

To represent the latent status of a gene (whether or not it is a cell cycle gene) we associate a hidden variable Ci with each gene node. Ci = 1 means that this gene is cell cycle regulated, otherwise Ci = 0. The joint probability distribution over the random variables Ci of this model is defined as

$$L = \frac{1}{Z} \prod_i \psi_i(C_i) \prod_{i,j} \psi_{ij}(C_i, C_j)$$

where ψ_i and ψ_{ij} are node and edge potential functions, and Z is the normalization term. Potential functions are defined to capture constraints on a single variable or between a pair of dependent variables. The edge potential function has the form $\psi_{ij}(C_i, C_j) = \exp\left\{-\lambda w_{ij}(C_i - C_j)^2\right\}$, where w_{ij} is the edge weight between two gene nodes *i* and *j*, and hyperparameter λ is a positive number. If two gene nodes are connected by an edge with a large weight, it is likely that they are functionally related. Thus, the potential function will penalize assignments that are different for the two nodes. The hyperparamter λ controls the relative contributions from sequence similarity and from gene expression, and can be tuned via cross validation.

A key to our algorithm is the derivation of an expression score consistent across all species used. Once such a score has been derived, each score node is assigned the corresponding gene's score, Si. We assume there are two score distributions for each species, one for cycling genes and the other for non-cycling genes, and Si is drawn from a mixture of these two. An important modeling issue is to choose the form of the two distributions. Because cycling score calculation involves taking the maximum peak of the expression time series or the Fourier transform and the resulting distribution often has a heavy tail, it turns out they can be better modeled as an Extreme Value Distribution (EVD) than a Gaussian distribution. An EVD is defined using two parameters: the location parameter and the scale parameter, similar to the mean and variance parameters of a Gaussian distribution. As a result we need to estimate four parameters for each species.

The model parameters are estimated using an EM-type algorithm. We start with an informative guess for the score parameters. Based on the score distributions we determine a posterior assignment to nodes using belief propagation. Following convergence of the belief propagation algorithm we use the (soft) label assignments to update the score distribution paramters. We repeat these steps by performing belief propagation again based on the updated score distributions and so forth until both the label assignment and score distribution parameters do not change any more.

Below we present a histogram detailing the increase (and decrease) in *rank* of genes between the initial scores (using expression alone) and the posterior scores derived by our algorithm (note that it is impossible to compare these scores directly as the units are different which is why we use rank). As can be seen the change approximately follows a normal distribution centered around 0, and the max change is less than 0.5 indicating that a gene with an initial score of 0 cannot be assigned a cycling status using our algorithm.

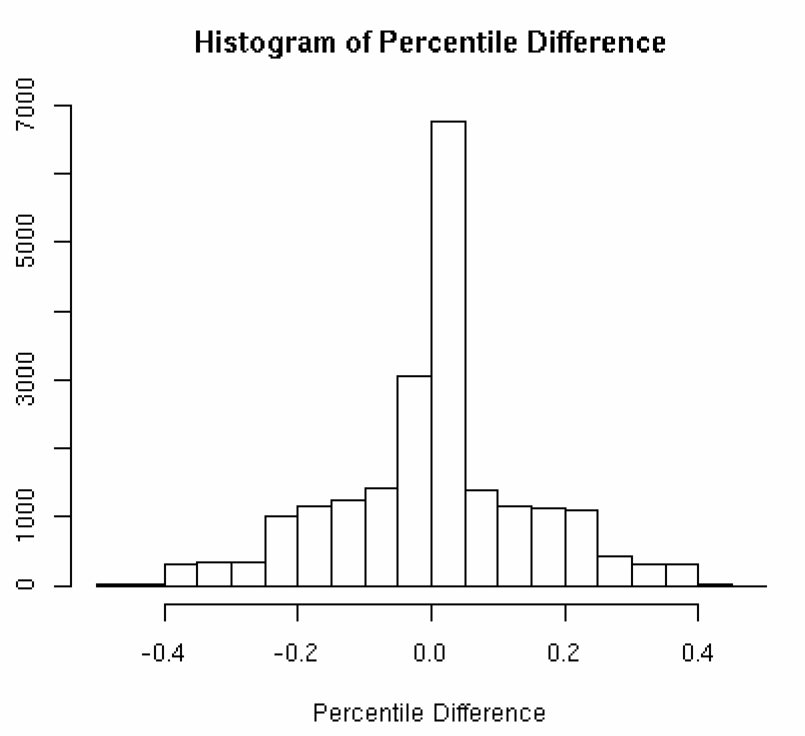

2. Sequence data and construction of homology graph

Non-redundant protein sequences were retrieved from ftp://ftp.ncbi.nlm.nih.gov/. We ran BLASTALL on these sequences with parameters similar to those used in INPARANOID [63]. E.g. to run BLASTALL between all human proteins and budding yeast proteins, we used the following command:

```
blastall -i BYEAST -d HUMAN -p blastp -v 6147  -b 23768 -F "m S" -M BLOSUM62 -z 5000000
```

where the numbers following –p and –v are the numbers of input proteins in the two species.

The output from BLASTALL was parsed to extract bit scores between pairs of proteins. We selected all pairs of genes whose bit score was above a threshold and used them to construct a homology graph. Each gene is represented by a node in the graph, and two genes are connected by an edge when their bit score is higher than the threshold, with the edge weight set to the bit score. We tried several different thresholds: 50, 75, 100, 150, 200, and 250. Our result is based on the threshold bit score of 100 because it is conservative enough to capture significant phylogenetic relations, while not throwing away too much information.

3. Graph partitioning

After estimating the posterior for all genes, we removed genes with lower posteriors from the graph using species specific thresholds. The remaining subgraph contained the top 500 arabidopsis genes, the top 800 budding yeast genes, the top 600 fission yeast genes, and the top

1000 human genes. We used the Markov clustering algorithm [14] to partition the remaining subgraph into densely connected groups. Specifically, we used the following command to partition the graph:

```
mcl -I 2.0 -pi 5.0
```

While most subgraphs had less than 50 genes, some of the resulting subgraphs had more. For these we further partition these subgraphs recursively until there were at most 50 genes in each of the resulting subgraphs. In the end, we divided all cycling genes (in all species) into groups based on the set of other species represented in the subgraph they belonged to. Genes in subgraphs with genes from all four species were assigned to the CCC4 sets. Notice that there are four CCC4 sets, one for each species. Similarly we created the CCC3 sets for budding yeast, fission yeast, and humans, and CCC2 sets for budding yeast and fission yeast.

4. Motif analysis

Outline:

This study analyses four collections of datasets:

1. ***S. pombe* cell-cycle conserved genes:** *S. pombe* cell-cycle genes in CCC2.
2. ***Negative control:*** Non-cell cycle *S. pombe* homologs of *S. cerevisiae* cell cycle genes.
3. ***Positive control:*** *S. cerevisiae* cell-cycle genes in CCC2.
4. ***Extended positive control:*** All *S. cerevisiae* cell-cycle genes (conserved and non-conserved).

Motif-Finding Methodology:

For each gene in the lists, the appropriate intergenic region was extracted from the *S. pombe* or *S. cerevisiae* genome. Four motif finders were run on each dataset; SOMBRERO [24,25], BioProspector [26], Consensus [27], and AlignACE [28]. Both SOMBRERO and BioProspector require a background model, and the background was constructed from all intergenic regions (in the appropriate genome) for both cases. SOMBRERO was run using default settings (iterating to find overrepresented motifs of lengths 8bp to 20bp on either strand), and simultaneously using all known *S. cerevisiae* motifs as an appropriate source of prior knowledge. Consensus and BioProspector were run using mostly default settings, but requiring the top 10 motifs to be reported. AlignACE was run using default settings (using a seed motif length of 10), and provided with the background intergenic GC-content (31.45%). The top 10 motifs from each method are reported in the attached documents for each of the datasets. Motifs are highlighted if they bear similarity to the known *S. cerevisiae* motif.

Percentage of genes identified by each motif-finder:

We examine the top 10 discovered motifs from each of the four motif-finders used in this study. The top 10 motifs from each motif-finder may contain zero to many matches against the “known” (Harbison/MacIsaac) motif [23,64]. In constructing these motifs, the motif-finding algorithms align a set of related sequences (i.e. potential transcription factor binding sites). This alignment implicitly incorporates some “threshold”, optimized by the algorithm, which decides which sequences to include and which to ignore for the purposes of constructing the motif. The four

motif-finders used in our study not only report the discovered motifs, but also the lists of sequences/sites that went into constructing these motifs.

For the recovered motifs that match the expected cell-cycle motif, we can take the lists of sites *provided by the motif-finders* as our set of binding site predictions. This allows us to forget about choosing a threshold as in the motif-scanning approach. Some of the sites in the list will be true transcription factor binding sites, but the lists will also usually include some non-functional spurious matches. This is because no motif-finder is perfect, and there is no existing computational measure for which potential sites are functional.

It would be very difficult to tell which sites are functional if we only had a single motif prediction (and a single list of sites). However, in most cases we have multiple discovered motifs that match the expected cell-cycle motif. Therefore, we can merge the lists of expected sites and find the sites that are predicted by more than one motif. Since these sites are recovered multiple times, we have greater confidence in their functionality. We still have to choose some threshold for how many motifs/lists a sites has to be in. This has to be an *ad hoc* choice, but no more so than choosing a threshold and scoring function in motif-scanning tests. In the below, we have required sites to be in at least 2 or at least one third (whichever is the greater number) of motif-lists.

The procedure is illustrated in Figure M1 below. For those sites that pass the threshold, we have counted the numbers of genes/promoters and these are reported in Table 1. In cases where the expected motif is recovered 0 or 1 times, we have put a “0%”.

Figure M1: Percentage analysis for motif finders

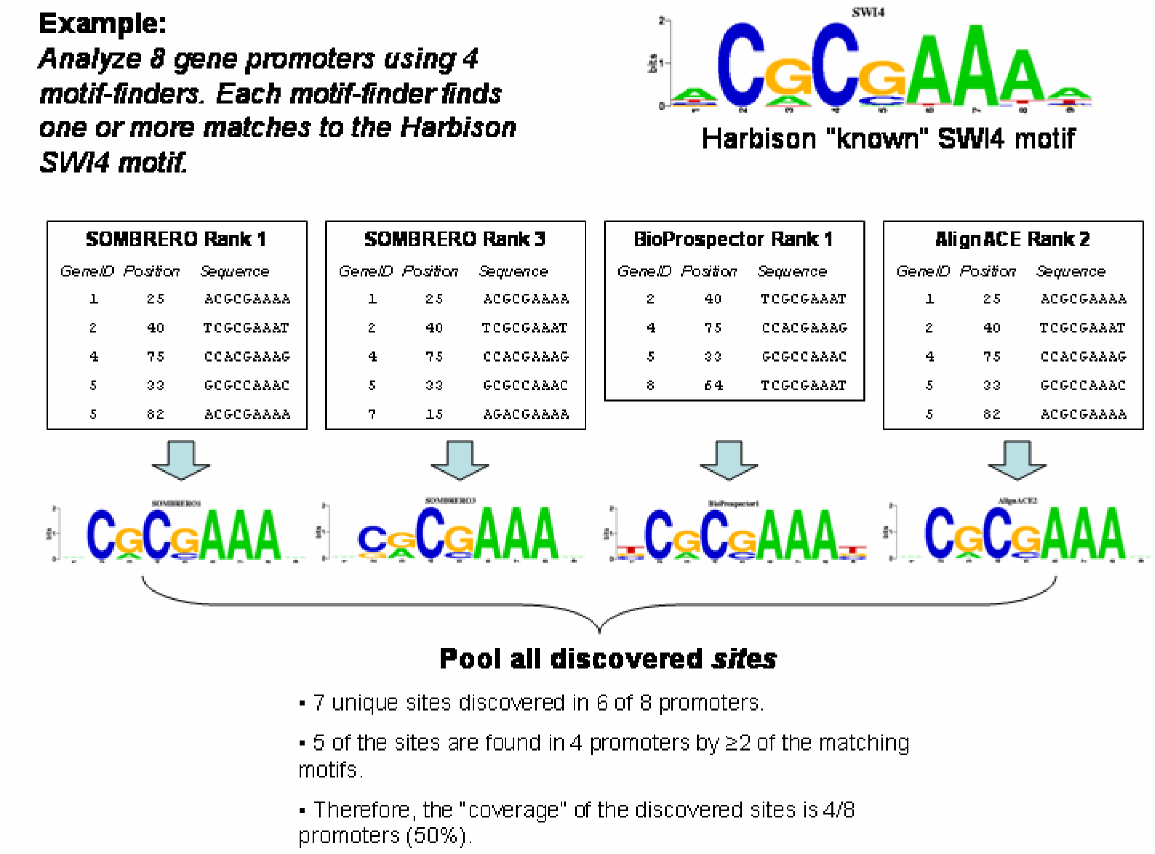

Motif-finding Results:

The results are shown in Table 1 in the main text and in Table M1 below.

In the G1/S phase, the SWI4, SWI6 and MBP1 datasets display the optimal motif conservation pattern; the expected motifs are found in both the *S. pombe* cell-cycle genes and the positive control of conserved *S. cerevisiae* cell-cycle genes (by all four motif-finders), but not in the negative control set of non-cell-cycle *S. pombe* genes.

In the G2/M phase, the FKH2 motif is conserved between *S. pombe* and *S. cerevisiae* cell-cycle datasets. However, the FKH2 motif also appears in the negative control FKH2 set. The FKH1 motif was not found in the *S. pombe* FKH1 cell-cycle dataset. In total, FKH-like motifs are present in 6 of the 11 *S. pombe* cell-cycle datasets and 4 of the 11 negative control datasets, suggesting that FKH motifs have widespread transcriptional control in *S. pombe* and that FKH motifs are necessary but not sufficient for G2/M-phase cell-cycle expression in *S. pombe*.

In terms of other transcriptional signals that are unique to *S. pombe* G2/M-phase expression, we still do not find any clear evidence of the PBF motif described by Bähler and others ("GNAACR"), and, as previously, the MCM1/NDD1 motif is absent from *S. pombe*. However, all *S. pombe* G2/M-phase cell-cycle datasets contain SWI4/6-like motifs, and the SWI4/6-like motifs are absent from the negative controls. In fact, the SWI4/6 motifs are also strongly supported in the FKH1 positive control datasets. It is also quite interesting that the "AGGGT" motif is now found in the *S. pombe* cell-cycle dataset and the positive controls but not in the negative controls. This motif is similar to the HIR1 (histone) binding motif in *S. pombe*.

Our M/G1-phase analysis is complicated by small dataset size. This is a particular problem in the positive control datasets, where the known *S. cerevisiae* motifs are not recovered until the sets are expanded to include all budding yeast cell-cycle genes. However, strong evidence exists that the ACE2 motif is present in the cell-cycle *S. pombe* dataset (all four motif-finders recover a similar motif). A weak match to the SWI5 motif is found by two of the motif-finders in the current study. Note also the presence of the previously mentioned "AGGGT" HIR1-like motif in the positive control SWI5 & ACE2 datasets.

In relation to the YOX1 motif the Harbison and MacIsaac predicted motifs are significantly different ("AATA" and "ATTAnnTTTCCnAAnnnGGG" resp.). YOX1 is a homeo-domain TF. In the MacIsaac predicted motif, the "ATTA" pattern appears in conjunction with a MCM1-like motif ("CCnAAnnnGGG"). It now seems that the positive controls strongly contain the MCM1-like motif, but not the "ATTA" motif. We therefore counted MCM1-like motifs as a correct matches for the YOX1 dataset. This would also explain why we cannot find a YOX1 motif in *S. pombe* cell cycle genes; no MCM1-like motif is present in any of the *S. pombe* datasets.

Table M1: Summary of motif-finding results. The number of motif-finders that recover the appropriate TF's motif is given for each set (0-4). Named TFs in parentheses represent other motifs found in the appropriate dataset.

	**Dataset**	***S. pombe* cell-cycle genes**		**Negative control (*S. pombe* non cell-cycle genes)**		**Positive control (conserved S. cerevisiae cell-cycle genes)**		**Extended positive control (all budding yeast CC genes)**	
**G1/S**	**SWI4**	4	(ACE2 & FKH)	0		4		4	
	**SWI6**	4	(FKH)	0	(FKH)	4		4	(MCM1)
	**MBP1**	4	(FKH)	0	(FKH)	4		4	(MCM1)
**G2/M**	**FKH1**	0	(SWI4/6)	2		1	(SWI4/6)	3	(SWI4/6)
	**FKH2**	2	(SWI4/6 & "AGGGT")	2		1	(MCM1 & "AGGGT")	2	(MCM1 & "AGGGT")
	**NDD1**	0	(SWI4/6 & FKH)	0	(FKH)	4	(SWI4/6)	4	
**M/G1**	**MCM1**	0	(SWI4/6)	0	(FKH)	3		4	
	**ACE2**	4*		0		0*	(SWI4/6)	4	(MCM1 & "AGGGT")
	**SWI5**	~2*	(FKH)	0		~2*		1	("AGGGT")
	**YOX1**	0*	(ACE2 & SWI4/6)	0*		3*		3	(SWI5)
	**YHP1**	0*	(ACE2)	0*		1*		~1*	("CAGTTG")

* These datasets contain ≤ 10 genes.

~ denotes weak matches to the known motif.

How many promoters in each cell-cycle dataset are the discovered motifs located in?

The easiest way to answer the above question is to examine the sites contributing to the motif-finders matches to the target motifs. In Supporting Table 4 (reproduced as Table M2 below), we present results in which all promoters were scanned using the appropriate Harbison motifs (but replacing the MCM1 motif for YOX1). In order to set a threshold, We generated 10Mbp of random sequence using the same GC-content as budding or fission yeast. We next set the threshold for each motif as that which gives a false discovery rate (FDR) of 0.0001 in these random datasets. The results support the conclusions for G1/S and for Fkh2.

Table M2: Percentage of input promoter datasets matching appropriate Harbison motif

Dataset	*Fission Cell Cycle*	*Fission Non-Cell Cycle*	*Budding Cell Cycle Conserved*	*Budding Cell Cycle All*
SWI4	29.73%	26.06%	60.71%	55.56%
SWI6	51.43%	30.46%	60%	50.54%
MBP1	52.27%	16.57%	51.72%	45.98%
FKH1	21.95%	25%	52.38%	36.23%
FKH2	32.50%	13.43%	39.13%	40%
NDD1	17.65%	31.48%	50%	44.90%
MCM1	26.92%	15.38%	73.33%	69.05%
ACE2	28.57%	29.63%	33.33%	48.48%
SWI5	50%	44.44%	25%	38%
YOX1	25%	12.50%	42.86%	57.14%
YHP1	0%	0%	50%	33.33%

5. Conservation analysis of protein complexes

Using data from Krogan *et al.* [18] and Gavin *et al.* [19], we determined protein complexes in budding yeast that are enriched with conserved cycling genes. We used the hypergeometric distribution to compute p-values for the enrichment. E.g. if a protein complex is composed of k genes and m of them are in the CCC4 set, then the p-value (for enrichment of CCC4 genes) is computed as the probability of finding at least m CCC4 genes when we randomly pick k genes from the set of all genes. We listed protein complex enriched with conserved cycling genes (pval < 0.05) in Supporting Table 12 and Supporting Figure 17.

6. Protein-protein interaction analysis

We analyzed the conserved cycling genes for enrichment of protein-protein interactions using data from Krogan *et al.* [18] and Gavin *et al.* [19] (both for budding yeast), and Rual *et al.* [65] (for humans). We considered two genes to interact with each other if they were listed in the same complex. We first counted the number of interactions between genes in the CCC3 set, and then compared it to that of random sets of cycling genes. Specifically, we randomly drew 1000 sets from the list of all cycling genes, all having the same size of the CCC3 set. For each random set, we counted the number of protein-protein interactions between genes in the set. Finally we plotted the empirical distribution of these numbers, and compared it with the number of interactions between CCC3 genes (See Figure 3 in main text).

7. GO analysis

The Gene Ontology (GO) enrichment analysis was performed by the STEM program [44]. For each list of genes, we determined the GO terms enriched for genes in that list. P-values for enrichment were computed using the hypergeometric distribution, with the list of all genes as the reference set. The GO terms were ranked by their p-values, from the lowest (most significant) to the highest (least significant). To determine the difference between functions of conserved cycling genes and general cycling genes, we compared the top 20 enriched GO terms in the CCC3 set and those in the list of all cycling genes. Specifically, we determined the GO terms enriched in top 20 for both the CCC3 set and the general set, as well as those in the top 20 for only one of the lists.

The results are presented in Supporting Tables 13-21.

8. Essentiality analysis

We retrieved the list of essential budding yeast gene from [66]. These genes have been determined to be essential for growth on rich glucose media. For each of the five lists in Figure 4 of the main text, we determined the percentage of genes that are essential. The five lists are:

1. cycling genes in Spellman *et al.* [2]
2. cycling genes in Spellman *et al.* with homologs in all other three species
3. our list of cycling genes
4. CCC3 – cycling budding yeast genes with cycling homologs in both fission yeast and humans
5. CCC4 – cycling budding yeast genes with cycling homologs in all other three species

For the second list, the homologs were determined by BLASTALL, using a cutoff bit score of 50. For each list, we also computed the p-value for the percentage using the hypergeometric distribution.

We analyzed lists of human cycling genes in a similar way, using human RNAi knockout data from [30]. The list of 1152 genes that were required for normal cell cycle progression was downloaded from the supporting website of [30]. For each of the following five lists, we determined the percentage of genes that are in the list by Mukherji et al.:

1. cycling genes in Whitfield *et al.* [1]
2. cycling genes in Whitfield *et al.* with homologs in all other three species
3. our list of cycling genes
4. CCC3 – cycling human genes with homologs in budding yeast and fission yeast
5. CCC4 – cycling human genes with homologs in all other three species

As before, the homologs in the second list were determined by BLASTALL using a cutoff bit score of 50.
